# Lsr2 and Its Novel Paralogue Mediate the Adjustment of Mycobacterium smegmatis to Unfavorable Environmental Conditions

**DOI:** 10.1128/mSphere.00290-21

**Published:** 2021-05-12

**Authors:** Marta Kołodziej, Tomasz Łebkowski, Przemysław Płociński, Joanna Hołówka, Mariola Paściak, Bartosz Wojtaś, Katarzyna Bury, Igor Konieczny, Jarosław Dziadek, Jolanta Zakrzewska-Czerwińska

**Affiliations:** a Department of Molecular Microbiology, Faculty of Biotechnology, University of Wrocław, Wrocław, Poland; b Institute of Medical Biology, Polish Academy of Sciences, Łódź, Poland; c Department of Immunology and Infectious Biology, Faculty of Biology and Environmental Protection, University of Łódź, Łódź, Poland; d Department of Immunology of Infectious Diseases, Hirszfeld Institute of Immunology and Experimental Therapy, Polish Academy of Sciences, Wrocław, Poland; e Laboratory of Molecular Neurobiology, Neurobiology Center, Nencki Institute of Experimental Biology, Polish Academy of Sciences, Warsaw, Poland; f Intercollegiate Faculty of Biotechnology of University of Gdansk and Medical University of Gdańsk, University of Gdańsk, Gdańsk, Poland; University of Iowa

**Keywords:** Lsr2, *Mycobacterium*, NAPs, lipooligosaccharides

## Abstract

Lsr2 is a nucleoid-associated protein (NAP) that has been found strictly in actinobacteria, including mycobacteria. It is a functional homolog of histone-like nucleoid-structuring protein (H-NS); it acts as a DNA-bridging protein that plays a role in chromosomal organization and transcriptional regulation. To date, the studies on Lsr2 have focused mainly on Mycobacterium tuberculosis. In this study, we analyze the role of Lsr2 as a transcription factor in Mycobacterium smegmatis, a saprophytic bacterium whose natural habitat (soil and water) substantially differs from those of the obligatory mycobacterial pathogens. Our chromatin immunoprecipitation-sequencing (ChIP-seq) data revealed that Lsr2 binds preferentially to AT-rich regions of the M. smegmatis chromosome. We found that Lsr2 acts mainly as a repressor, controlling gene expression either directly by binding promoter regions or indirectly through DNA loop formation and DNA coating. One of the Lsr2-repressed genes encodes polyketide synthase (MSMEG_4727), which is involved in the synthesis of lipooligosaccharides (LOSs). An M. smegmatis strain deprived of Lsr2 produces more LOSs, which is mirrored by changes in the smoothness of cells and their susceptibilities to antibiotics. Unlike M. tuberculosis, M. smegmatis additionally encodes a paralogue of Lsr2, MSMEG_1060, which is a novel member of the mycobacterial NAP family. The Lsr2 and MSMEG_1060 proteins exhibit different DNA binding specificities and chromosomal localizations. Our results suggest that these proteins help M. smegmatis cells cope with stress conditions, including hypoxia and exposure to antibiotics. Thus, the present work provides novel insight into the role of Lsr2 paralogues in the ability of a saprophytic mycobacterial species to adjust to environmental changes.

**IMPORTANCE** Nucleoid-associated proteins (NAPs) are the most abundant proteins involved in bacterial chromosome organization and global transcription regulation. The mycobacterial NAP family includes many diverse proteins; some are unique to actinobacteria, and many are crucial for survival under stress (e.g., HupB and Lsr2) and/or optimal growth conditions (e.g., mycobacterial integration host factor [mIHF]). Here, we present a comprehensive study concerning two functional homologues of mycobacterial H-NS: Lsr2 and its paralogue from M. smegmatis, MSMEG_1060. We found that Lsr2 plays a role in transcriptional regulation, mainly by repressing gene expression via DNA loop formation and/or DNA-coating mechanisms. Intriguingly, the number of Lsr2-mediated genes was found to increase under hypoxia. Compared to Lsr2, MSMEG_1060 exhibits a different DNA binding specificity and chromosomal localization. Since tuberculosis remains a serious worldwide health problem, studies on stress response-mediating agents, such as Lsr2, may contribute to the development of novel antituberculosis drugs.

## Abstract (precis)

Nucleoid-associated proteins (NAPs) are the most abundant proteins involved in bacterial chromosome organization and global transcription regulation. The mycobacterial NAP family includes many diverse proteins; some are unique to actinobacteria, and many are crucial for survival under stress (e.g., HupB and Lsr2) and/or optimal growth conditions (e.g., mycobacterial integration host factor [mIHF]).

## INTRODUCTION

The genus Mycobacterium comprises a highly diverse group of organisms encompassing both environmental saprophytes (e.g., M. smegmatis) and pathogens, the latter of which include some species that cause serious diseases in mammals (e.g., M. tuberculosis and M. bovis). These Gram-positive, aerobic bacteria are surrounded by a unique cell envelope consisting of the inner plasma membrane, the peptidoglycan-arabinogalactan complex, and a peculiar outer membrane frequently termed the “mycomembrane” (1, 2).

So far, almost 200 mycobacterial species have been described (3). They inhabit a wide range of environments, including water, soil, and various niches within human and animal hosts (e.g., lung alveolar macrophages). Mycobacteria have evolved a plethora of strategies to facilitate their survival under various (frequently harsh) environmental conditions, including low oxygen levels (4).

To survive, mycobacteria (like other microorganisms) must quickly adapt to changing environmental conditions. Among the most rapid and effective adaptation strategies are changes in the transcriptional landscape mediated by global transcription factors. Nucleoid-associated proteins (NAPs) organize the chromosomal structure and act as global transcription regulators (5). They are believed to play crucial roles in the ability of a bacterium to quickly adapt to unfavorable conditions, particularly under stress (6). By inducing topological and/or structural changes in DNA, they can affect the global transcription profile.

Mycobacteria possess a unique set of NAPs, some of which are involved in the pathogenicity of tubercle bacilli (5). These NAPs include HupB, mycobacterial integration host factor (mIHF), and Lsr2, which are structural or functional homologs of the well-known Escherichia coli proteins HU, IHF, and histone-like nucleoid-structuring protein (H-NS), respectively; of them, some exhibit unique features (e.g., HupB, Lsr2) and/or are essential (e.g., mIHF) (7, 8). Recently, two novel mycobacterial NAPs were identified; NapM is a conserved protein in mycobacteria, while MSMEG_1060 is a putative paralogue of Lsr2 that is found in M. smegmatis but absent from M. tuberculosis (9, 10). While NapM has been relatively well characterized (9, 11), almost no data are available regarding the function of MSMEG_1060, except that immunoprecipitation of FLAG-tagged proteins suggested that it may be associated with the chromosome (10).

Lsr2, one of the principal mycobacterial NAPs, is highly conserved throughout the *Actinobacteria*, including *Corynebacterium* (12) and the antibiotic-producing *Streptomyces* (13). Lsr2 may play both architectural and regulatory roles and is believed to function similarly to H-NS; it preferentially binds AT-rich sequences and is able to either bridge distant DNA fragments or form a rigid nucleoprotein filament (14, 15). Like its H-NS counterpart in the *Proteobacteria* (16), Lsr2 appears to act as a xenogeneic silencer of the AT-rich regions that are frequently acquired by horizontal gene transfer (13, 17, 18).

In mycobacteria, the regulatory role of Lsr2 has previously been studied mainly in pathogenic species, such as M. tuberculosis (19). In this setting, Lsr2 has been shown to be (i) crucial during infection, (ii) required for virulence and adaptation to changing oxygen levels (19), and (iii) able to downregulate the expression levels of major virulence factor-encoding genes (17). Recent work demonstrated that in M. tuberculosis, Lsr2 activity is modulated by protein kinase B (PknB), which controls the adaptation of M. tuberculosis to changing conditions inside host cells (20).

In M. smegmatis, deletion of the *lsr2* gene does not affect planktonic growth under optimal conditions (21). However, a lack of Lsr2 appears to profoundly impact the colony morphology of this saprophyte, leading to the formation of round and smooth colonies and the inability to form biofilm (22). We recently showed (23) that M. smegmatis cells deprived of Lsr2 are shorter and more rigid than wild-type (WT) cells, that Lsr2 forms large and dynamic nucleoprotein complexes *in vivo*, and that deletion of *lsr2* exerts a profound effect on the replication time and replisome dynamics. Moreover, we demonstrated that the N-terminal oligomerization domain of Lsr2 is indispensable for the formation of nucleoprotein complexes *in vivo*. Collectively, previous studies focusing on Lsr2’s role in both M. smegmatis and M. tuberculosis indicated that this protein exerts pleiotropic effects on cellular processes and appears to be an interesting target for the development of novel antitubercular drugs.

In the present study, we sought to explore the role of Lsr2 as a regulator of transcription in the saprophytic species M. smegmatis. We used RNA sequencing (RNA-seq) to compare the global transcription profiles of *Δlsr2* and wild-type M. smegmatis strains in an attempt to decode the Lsr2 regulatory network under optimal conditions or during hypoxia. We show that Lsr2 binds AT-rich regions and acts mainly as a gene repressor, either directly by binding promoter regions or indirectly through DNA loop formation. The most profound effect of *lsr2* deletion was the activation of *MSMEG_4727*, which encodes a putative polyketide synthase involved in the synthesis of the mycobacterial outer membrane component lipooligosaccharides (LOSs). Finally, we present a preliminary characterization of MSMEG_1060, which, in contrast to its paralogue Lsr2, occupies the entire nucleoid.

## RESULTS

### The Lsr2 regulon includes genes that are scattered throughout the genome but grouped within AT-rich regions.

**FIG 1:**
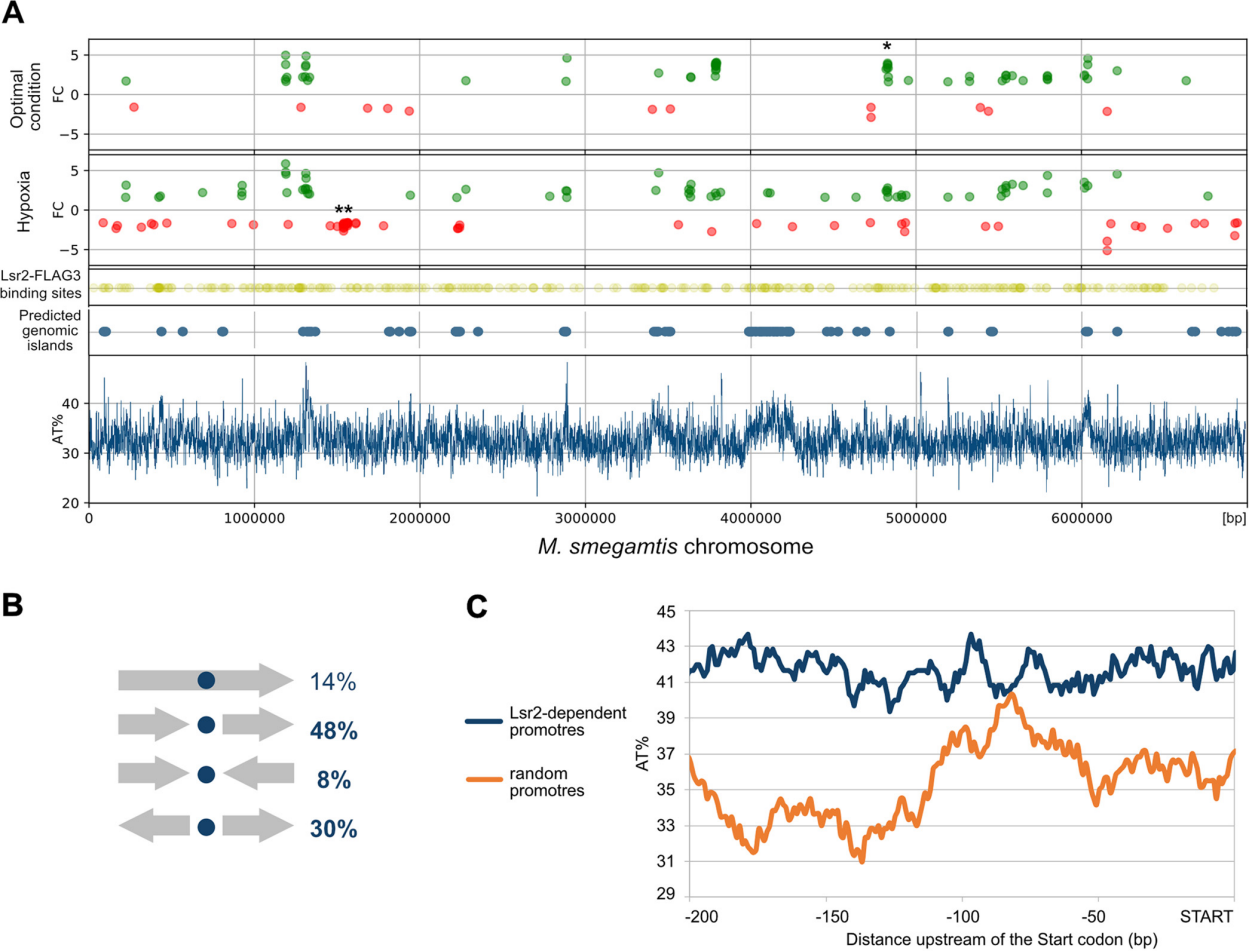
Regulatory properties of Lsr2. (A) Dot plots present M. smegmatis genes upregulated (green) and downregulated (red) in the Δ*lsr2* strain under two conditions (optimal and hypoxia). Yellow dots present Lsr2-FLAG_3_ binding sites determined by ChIP-seq. Some of the gene clusters correspond to predicted genomic islands visualized as blue dots (Island Viewer 4; https://www.pathogenomics.sfu.ca/islandviewer). The bottom panel presents the distribution of AT base pairs on the M. smegmatis chromosome (the calculation of average AT percentages of sequences in 500-bp windows, with a 500-bp step). Most of the regulated genes are grouped in AT-rich chromosome regions. *, MSMEG_4727-35 cluster; **, rp genes. (B) Orientation of Lsr2-FLAG_3_ binding sites in relation to M. smegmatis genes. (C) Analysis of the average AT contents in the promoter regions of genes regulated by Lsr2 (dark-blue line) and randomly chosen promoters (orange line) (*n* = 50).

To determine the complete Lsr2 regulon in M. smegmatis, we compared global transcript levels of the WT strain with those of the *lsr2* deletion mutant. Our RNA-sequencing (RNA-seq) analysis revealed that, under optimal growth conditions, Lsr2 regulates only 71 genes with a log_2_ fold change (FC) greater than 1.585 (which indicates that the level of transcription increased or decreased at least 3 times [see Table S1 in the supplemental material and GEO database accession number GSE169318]) and, in the majority of these cases, downregulates gene expression (59 genes). Notably, 51 of the Lsr2-regulated genes (72%) are predicted to have their own promoter elements (MicrobesOnline Operon Predictions, http://www.microbesonline.org). To reveal the possible regulatory mode(s) of Lsr2, we examined the DNA binding sites of Lsr2 along the M. smegmatis chromosome using chromatin immunoprecipitation-sequencing (ChIP-seq) and then combined the global map of Lsr2 binding sites with our RNA-seq data (Fig. 1A). The ChIP-seq experiments were performed using an M. smegmatis strain producing Lsr2 fused with three repeats of the FLAG epitope (Lsr2-FLAG_3_) and the WT strain lacking the FLAG epitope as a negative control (for experimental details, see Materials and Methods). The Lsr2-FLAG_3_ strain exhibited a growth rate and colony morphology (a rough colony morphotype) similar to those of the WT strain, and the presence of the fusion proteins in cell extracts was confirmed by Western blotting (Fig. S1). We performed two independent biological replicates; 583 sites were identified in both replicates and were evenly distributed along the M. smegmatis chromosome (Fig. S2A). Based on the ChIP-seq library containing Lsr2-FLAG_3_ binding sites (GEO database accession number GSE164708), we found that the majority (86%) of the Lsr2 binding sites are located within intergenic regions, i.e., in tandem, convergent, or divergent gene pair architecture (Fig. 1B). We analyzed the sequences of the DNA fragments bound by Lsr2 using the MEME and RSAT suites but failed to identify any consensus motif. Integrative analysis of whole-genome RNA-seq and ChIP-seq data showed that 31 of the Lsr2-regulated genes contained Lsr2 binding sites within their putative promoter regions (binding site within 150 bp upstream of the start codon); thus, these genes are presumed to be directly regulated by Lsr2. Our RNA-seq data indicated that almost all of these genes were repressed (28 repressed, 3 activated [Table S1]). Most of the identified genes have not been previously characterized and encode hypothetical proteins. A few encode proteins that have been predicted to be involved in different cellular processes, including *MSMEG_1060*, which encodes a novel Lsr2-like NAP, and *MSMEG_4727*, which encodes a mycocerosic acid synthase thought to be involved in the synthesis of LOS (see below for additional details on both).

10.1128/mSphere.00290-21.1 FIG S1 Phenotypic analysis of the M. smegmatis strains. Growth curves of the wild-type (WT), Δ*lsr2*, and Δ*MSMEG_1060* strains under optimal growth conditions (A) and with oxygen depletion (B). (C) Western blot analysis of Lsr2-FLAG_3_ (under the optimal condition and during hypoxia) and MSMEG_1060-mTurquoise2 strains. Equal amounts of total proteins were loaded in the lanes. Monoclonal anti-FLAG M2 antibody produced in mouse (Sigma Aldrich/Merck; dilution 1:5,000) was used to detect the Lsr2-Flag3 protein in the analyzed strains, followed by goat anti-mouse IgG secondary antibody, conjugated with horseradish peroxidase (HRP, dilution 1:5,000; Invitrogen). Monoclonal anti-GFP antibody produced in mouse (Sigma Aldrich/Merck, dilution 1:10,000) was used to detect the MSMEG_1060-mTurquoise2 protein in the analyzed strains, followed by donkey anti-mouse IgG secondary antibody, conjugated with HRP (Invitrogen; dilution 1:10,000). Download FIG S1, TIF file, 0.6 MB. Copyright © 2021 Kołodziej et al. 2021 Kołodziej et al. https://creativecommons.org/licenses/by/4.0/ This content is distributed under the terms of the Creative Commons Attribution 4.0 International license.

10.1128/mSphere.00290-21.2 FIG S2 Analysis of Lsr2 binding sites on the M. smegmatis chromosome. (A) Distribution of Lsr2-FLAG_3_ binding sites overlapping in two independent biological repeats. (B) Distribution of distances between the two closest Lsr2 binding sites on the M. smegmatis chromosome and the lengths of ChIP-seq fragments for Lsr2-FLAG_3_ protein. The first quartile (Q1) is defined as the middle number between the smallest number and the median of the data set. The second quartile (Q2) is the median of a data set. The third quartile (Q3) is the middle value between the median and the highest value of the data set. Download FIG S2, TIF file, 0.7 MB. Copyright © 2021 Kołodziej et al. 2021 Kołodziej et al. https://creativecommons.org/licenses/by/4.0/ This content is distributed under the terms of the Creative Commons Attribution 4.0 International license.

10.1128/mSphere.00290-21.7 TABLE S1 Genes with altered expression in the Δ*lsr2* strain compared to their expression in the WT under the optimal growth condition. Blue-highlighted genes are bounded by Lsr2 in the promoter region. Download Table S1, DOCX file, 0.03 MB. Copyright © 2021 Kołodziej et al. 2021 Kołodziej et al. https://creativecommons.org/licenses/by/4.0/ This content is distributed under the terms of the Creative Commons Attribution 4.0 International license.

Since Lsr2 exhibits increased affinity toward AT-rich sequences *in vitro*, as previously reported (17, 18) and confirmed here by gel electrophoresis mobility shift assay (EMSA) (see Fig. 3; Fig. S4C and D), we compared the AT contents of the gene promoters directly regulated by Lsr2 with those of randomly chosen M. smegmatis promoters (*n* = 50) (Fig. 1C). As expected, the Lsr2-dependent promoters exhibited a higher AT content (nearly 6%; *P* < 0.00001) than the randomly selected promoters. The AT distribution along the promoters of Lsr2-regulated genes also differed from that of the randomly selected promoters; in contrast to the randomly selected promoters, the Lsr2-dependent promoters exhibited a regular distribution of AT.

10.1128/mSphere.00290-21.4 FIG S4 Characterization of the Lsr2 and MSMEG_1060 proteins. (A and B) Purification of Lsr2_His6_ and MSMEG_1060_His6_. Polyacrylamide gels presenting Lsr2_His6_ (A) and MSMEG_1060_His6_ (B) purification steps. M, PageRuler unstained protein ladder (Thermo Scientific). (C and D) DNA binding properties of Lsr2 and the MSMEG_1060 protein. An electrophoretic mobility shift assay (EMSA) showed the binding of both MSMEG_1060_His6_ and Lsr2_His6_ to two DNA fragments containing the promotor region of the *lsr2* gene (C) and the promotor region of the *MSMEG_1060* gene (D). (E) Amino acid sequences and secondary structures of Lsr2-like proteins. The comparison of M. smegmatis Lsr2-like (MSMEG_1060) and Lsr2 (MSMEG_6092) with Lsr2 from other Mycobacterium species; M. bovis (Mb3628c), M. leprae (ML0234), M. marinum (MMAR_5101), and M. tuberculosis (Rv3597c). The blurry red rectangle presents the characteristic RGR motif. Download FIG S4, TIF file, 2 MB. Copyright © 2021 Kołodziej et al. 2021 Kołodziej et al. https://creativecommons.org/licenses/by/4.0/ This content is distributed under the terms of the Creative Commons Attribution 4.0 International license.

**FIG 2:**
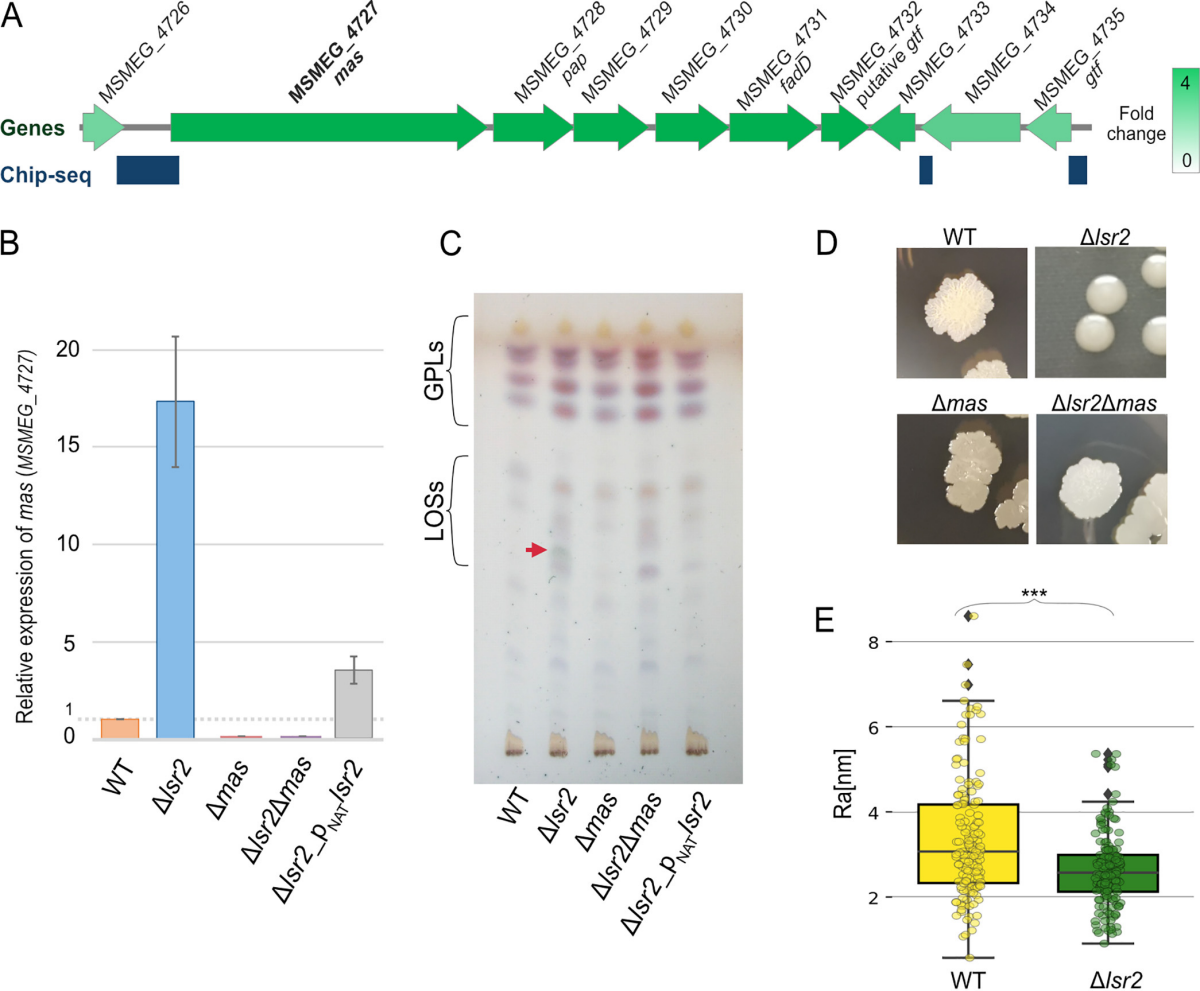
Determination of *MSMEG_4727* (*mas*) transcription levels using RT-qPCR and TLC analysis of lipooligosaccharides (LOSs) and glycolipids (GLPs). (A) Schematic of genes involved in LOS synthesis. The intensity of green represents the increase of gene expression in the Δ*lsr2* strain. Dark-blue rectangles correspond to the Lsr2-Flag_3_ binding sites. (B) The relative transcription of the *MSMEG_4727* gene (encoding a mas-like polyketide synthase [PKS] involved in LOS synthesis) in the wild-type (WT) M. smegmatis mc^2^ 155 strain, the *Δlsr2* mutant, the complemented strain (C; Δ*lsr2*_p_NAT_*lsr2*), the *Δmas* mutant, and the *Δlsr2 Δmas* double mutant, as assessed using RT-qPCR. (C) TLC analysis of crude methanol-soluble lipid fractions, as assessed from 100 μg of each sample. The solvent system was chloroform-methanol (90:10, vol/vol). Detection was at 0.2% anthrone in concentrated sulfuric acid. The red arrow indicates an LOS that is overrepresented in the Δ*lsr2* mutant. (D) Colony morphologies of the WT and Δ*lsr2* strains. (E) The Ra parameter (arithmetic mean deviation of the roughness profile) was calculated using AFM measurements taken from three or four 500-nm lines per cell (*n* = 50, bottom panel; statistical significance [***] was defined as a *P *of <0.0005, as determined by a parametric double-sided *t* test with pooled standard deviations).

Lsr2, like its structural homolog H-NS, may not only directly regulate gene expression but also indirectly affect gene expression through forming a DNA loop that bridges the upstream and downstream regions of a selected gene(s) (24, 25). To explore this possibility, we first estimated the distances between neighboring Lsr2 binding sites and then analyzed the locations of adjacent binding sites in relation to the chromosomal positions of the Lsr2-regulated genes. The median distance between the two closest Lsr2 binding sites was 5,973 bp, and the midspread (the interquartile range) ranged from 1,933 to 15,390 bp (Fig. S2B). Thus, Lsr2 may bridge DNA fragments consisting of 2 to as many as 16 genes (assuming that the average length of an M. smegmatis gene is 946 bp [https://mycobrowser.epfl.ch]). Further analysis revealed an interesting example of a set of nine genes (*MSMEG_4727* to *_4735*, ∼17 kb) that are presumably not organized into a single operon (Martini et al. [26] and MicrobesOnline Operon Predictions [http://www.microbesonline.org]), but the transcription of all of them was found to be significantly increased in the strain lacking Lsr2 (Fig. 2A). Our ChIP-seq analysis revealed that the set of genes is flanked by Lsr2 binding sites (Fig. 2A). Thus, these genes are repressed probably by binding promoter regions (or eventually through Lsr2-mediated DNA loop formation).

Together, our combined RNA-seq and ChIP-seq data revealed that Lsr2 controls gene expression either directly by binding promoter regions or indirectly by DNA loop formation.

### Lsr2-mediated repression of the polyketide synthase gene results in the rough colony morphology.

Our RNA-seq analysis revealed that the *MSMEG_4727* gene, which encodes a putative polyketide synthase (mycocerosic acid synthase-like polyketide synthase, *mas*), was one of the most significantly repressed genes by Lsr2 (FC = 3.2). To validate these RNA-seq results, we performed quantitative reverse transcription-PCR (RT-qPCR) using primers specific for *MSMEG_4727* (Table S3). Our results confirmed that the transcription level of *MSMEG_4727* was 17-fold higher in the Δ*lsr2* strain than in the WT strain (Fig. 2B). It has been demonstrated that MSMEG_4727 is likely to be involved in the synthesis of LOSs (27), which are components of the mycobacterial outer membrane (28). Interestingly, the pathogenic nontuberculous species (Mycobacterium abscessus and Mycobacterium kansasii) exhibit either a smooth (S) or a rough (R) colony morphotype, depending on the level of surface-associated glycolipids (glycopeptidolipid [GPL] or LOS), and the rough variants are more virulent than the corresponding smooth strains (29–31). Given that M. smegmatis lacking Lsr2 forms smooth colonies (21, 22) and might produce larger amounts of LOSs, we analyzed the cell surface of the Δ*lsr2* strain. For this purpose, atomic force microscopy (AFM) analysis was performed using the PeakForce Tapping mode (for details, see Text S1). The AFM analysis showed that the cell surface of the Δ*lsr2* strain was smoother than that of the WT strain; the calculated Ra parameters (arithmetic mean deviations of the roughness profile) were 3.33 ± 1.81 and 4.68 ±1.25 for Δ*lsr2* and WT cells, respectively (50 cells were analyzed for each strain) (Fig. 2E).

10.1128/mSphere.00290-21.9 TABLE S3 Strains, plasmids, and oligonucleotides used in this study. Download Table S3, DOCX file, 0.04 MB. Copyright © 2021 Kołodziej et al. 2021 Kołodziej et al. https://creativecommons.org/licenses/by/4.0/ This content is distributed under the terms of the Creative Commons Attribution 4.0 International license.

10.1128/mSphere.00290-21.10 TEXT S1 Experimental procedures; M. smegmatis mc^2^ 155 mutant strain construction; DNA manipulations, bacterial strains, and culture conditions; protein purification; DNA binding assay; atomic force microscopy (AFM); RNA isolation; reverse transcription and quantitative PCR (RT-qPCR); extraction of lipids and TLC analysis. Download Text S1, DOCX file, 0.1 MB. Copyright © 2021 Kołodziej et al. 2021 Kołodziej et al. https://creativecommons.org/licenses/by/4.0/ This content is distributed under the terms of the Creative Commons Attribution 4.0 International license.

Next, we examined whether MSMEG_4727 is responsible for the synthesis of LOSs and thus the smooth colony morphology. For this purpose, we constructed Δ*mas* strains in the WT and in the Δ*lsr2* background (for details, see Table S3 and Text S1). We examined the presence of LOSs in the WT, Δ*lsr2*, Δ*mas*, and the double-deletion Δ*lsr2* Δ*mas* strains using thin-layer chromatography (TLC) (for details, see Text S1). First, we checked if deletion of *MSMEG_4727* in the Δ*lsr2* strain restored a wild, rough colony morphology. Indeed, colonies of the Δ*lsr2*Δ*mas* double mutant differ from those of the Δ*lsr2* mutant, and its phenotype resembles that of the wild-type strain (Fig. 2D). The TLC analysis of methanol-soluble crude lipids revealed that there was an additional glycolipid spot in the Δ*lsr2* strain that was not observed in the WT, Δ*mas*, and Δ*lsr2*Δ*mas* strains (Fig. 2C). It should be noted that we did not observe any significant difference in the levels of polar lipids and mycolic acids among the analyzed strains (Fig. S3).

10.1128/mSphere.00290-21.3 FIG S3 Lipid analysis of the WT M. smegmatis mc^2^155 strain, *Δlsr2* mutant, *Δlsr2* complemented strain, *Δmas* mutant, and *Δlsr2 Δmas* strain performed by TLC. (A) TLC analysis of polar glycolipids of the WT M. smegmatis mc^2^155 strain, Δ*lsr2* mutant, Δ*mas* mutant, Δ*lsr2* Δ*mas* mutant, and Δ*lsr2* complemented strain (Δ*lsr2p MV306_lsr2)*. One hundred micrograms of each lipid extract was applied. The solvent system was chloroform-methanol-water (60:30:6, vol/vol/vol). Detection was with orcinol reagent. (B) TLC analysis of mycolic acid methyl esters fractions of the WT M. smegmatis mc^2^ 155 strain, Δ*lsr2* mutant, Δ*mas* mutant, Δ*lsr2* Δ*mas* double mutant, and the Δ*lsr2* complemented strain (Δ*lsr2p MV306_lsr2*). Three hundred micrograms of each sample was applied. The solvent system was hexane-diethyl ether (85:15, vol/vol). Detection was with 10% molybdophosphoric acid in ethanol. Download FIG S3, TIF file, 1.2 MB. Copyright © 2021 Kołodziej et al. 2021 Kołodziej et al. https://creativecommons.org/licenses/by/4.0/ This content is distributed under the terms of the Creative Commons Attribution 4.0 International license.

Together, these findings indicate that the rough colony morphology of M. smegmatis relies on the ability of Lsr2 to repress the expression of the *MSMEG_4727* gene (and other genes from this cluster).

### MSMEG_1060, a novel mycobacterial Lsr2-like NAP, colocalizes with the chromosome.

**FIG 3:**
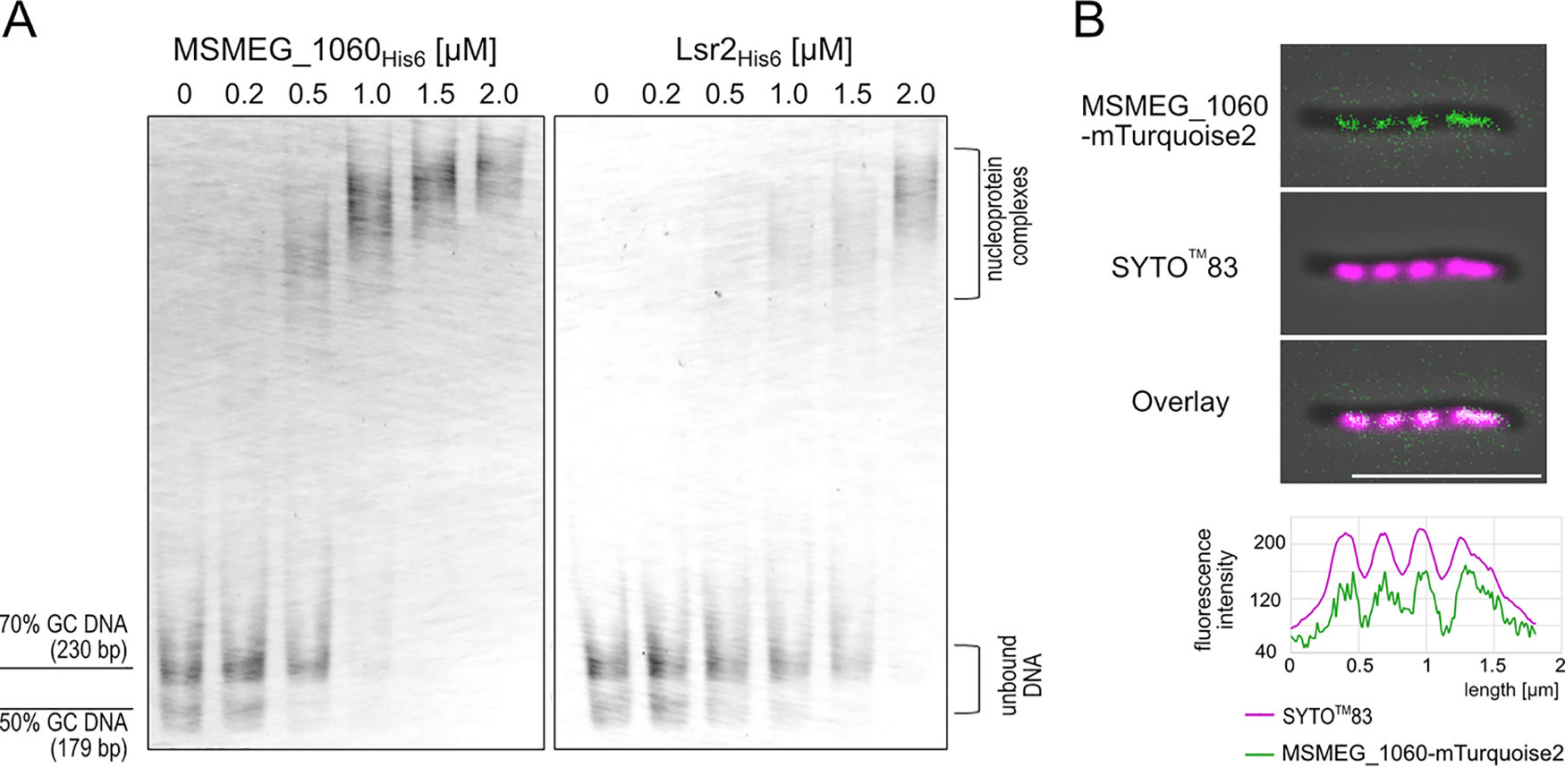
DNA binding properties of MSMEG_1060 protein. (A) Electrophoretic mobility shift assay (EMSA) showing the affinities of both MSMEG_1060_His6_ (left) and Lsr2_His6_ (right) to two DNA fragments that differed in their GC contents (50% and 70%). (B) Subcellular localization of MSMEG_1060-mTurquoise2 in M. smegmatis cells. The MSMEG_1060-mTurquoise2 fluorescence pattern was compared to that of the SYTO 83-stained nucleoid.

Since MSMEG_1060 was recently identified as an Lsr2-like protein (10) and our RNA-seq analysis demonstrated that Lsr2 downregulates the expression of the MSMEG_1060, we sought to investigate the function of MSMEG_1060 in M. smegmatis. Using RT-qPCR (for details, see Text S1), we confirmed (Fig. S5) that the *MSMEG_1060* gene is indeed downregulated by Lsr2, as the transcription level of *MSMEG_1060* was 2.6-fold higher in the Δ*lsr2* strain than in the WT. Our ChIP-seq analysis showed that Lsr2 appears to bind the putative promoter region of *MSMEG_1060*. The interaction of Lsr2 with the *MSMEG_1060* promoter region was additionally confirmed by EMSA (for details, see Text S1 and Fig. S4A to D), which showed that Lsr2 retarded the mobility of the DNA fragment encompassing the entire *MSMEG_1060* promoter region. Given that Lsr2 and MSMEG_1060 exhibit rather high amino acid similarity (54% similarity and 34% identity [Fig. S4E]), but mainly within the N-terminal domain (which is responsible for dimerization in Lsr2 [15]), we decided to examine whether MSMEG_1060 binds DNA *in vitro*. For this purpose, C-terminally six-histidine-tagged MSMEG_1060 protein was expressed in E. coli and then purified using affinity chromatography (as with Lsr2_His6_; for details, see Text S1 and Fig. S4A and B). Since Lsr2 exhibits a high affinity to AT-rich DNA (17, 18), we performed our EMSA using the DNA fragments DNA_50%_ (170 bp) and DNA_70%_ (230 bp), which had GC contents of 50% and 70%, respectively. The binding of MSMEG_1060_His6_ to both DNA fragments retarded the migration of DNA compared with that of free, unbound DNA, and as with Lsr2_His6_, MSMEG_1060_His6_ preferred the DNA fragment with the higher AT content (Fig. 3A). We observed a few DNA bands with decreased mobility for MSMEG_1060_His6_, suggesting that this protein, like Lsr2, may oligomerize via its N-terminal domain upon DNA binding (Fig. 3A). Notably, MSMEG_1060_His6_ bound both AT- and GC-rich DNA fragments with a higher affinity than Lsr2 (Fig. 3A). Thus, further comprehensive DNA-protein analyses are warranted to shed light on the differences between MSMEG_1060 and Lsr2 in terms of their DNA binding affinity and specificity.

10.1128/mSphere.00290-21.5 FIG S5 RT-qPCR analysis of the *lsr2* and *MSMEG_1060* genes’ relative expression levels. Bar plots presenting relative levels of transcription of the *lsr2* (left) and *MSMEG_1060* (right) genes under different stress conditions (NDX, 3 h of exposure to nalidixic acid at 50 mg/liter; RIF, 3 h of exposure to rifampicin at 5 mg/liter; HYP, 3 h of induced hypoxia, according to the RAD model) or in the M. smegmatis Δ*MSMEG_1060* Δ*lsr2* deletion strain. The presented respiratory quotient (RQ) values are means from three independent biological repeats. Gene expression was calculated relative to that in the reference sample (Δ*MSMEG_1060* Δ*lsr2* expression in the wild-type M. smegmatis strain under the optimal growth condition). Download FIG S5, TIF file, 0.2 MB. Copyright © 2021 Kołodziej et al. 2021 Kołodziej et al. https://creativecommons.org/licenses/by/4.0/ This content is distributed under the terms of the Creative Commons Attribution 4.0 International license.

Since our preliminary *in vitro* experiments suggested that MSMEG_1060 has a DNA binding affinity and specificity different from those of Lsr2, we decided to analyze its subcellular localization. We thus constructed an M. smegmatis strain that produces an MSMEG_1060-mTurquoise2 fusion protein from its native promoter at the endogenous chromosomal locus (for details, see Table S2). The MSMEG_1060-mTurquoise2 strain exhibited a colony morphology and growth rate similar to those of the WT (Fig. S1A and B; also data not shown), and the presence of the fusion protein was verified by Western blotting (Fig. S1C). Fluorescence microscopy revealed that MSMEG_1060-mTurquoise2 protein was visible as moderately bright, evenly distributed foci whose localization resembled that of the HupB protein, which is used as a chromosome marker in mycobacterial cells (7). Further analysis using the nucleic acid dye SYTO 83 confirmed that MSMEG_1060-mTurquoise2 did indeed colocalize with the nucleoid, although the foci were more blurred than the fluorescence signals of SYTO 83 (Fig. 3B, and see Fig. 5C). Thus, the localization of MSMEG_1060-mTurquoise2 substantially differed from that of Lsr2, which was visible as one or two discrete and bright major foci (see Fig. 5C) (23).

10.1128/mSphere.00290-21.8 TABLE S2 Gene expression during hypoxia. Download Table S2, DOCX file, 0.04 MB. Copyright © 2021 Kołodziej et al. 2021 Kołodziej et al. https://creativecommons.org/licenses/by/4.0/ This content is distributed under the terms of the Creative Commons Attribution 4.0 International license.

Taken together, our observations show that MSMEG_1060 is a novel NAP that binds AT- and GC-rich DNA fragments with a high affinity and, in contrast to Lsr2, occupies the entire nucleoid. Moreover, the gene transcription of *MSMEG_1060* is directly controlled by Lsr2.

### The Lsr2 regulon is altered by oxygen depletion.

Since previous findings demonstrated that the M. tuberculosis *Δlsr2* mutant is more susceptible to a change in oxygen level than are WT cells (19), we decided to examine whether oxygen depletion alters the gene expression profile of saprophytic mycobacterial species (i.e., M. smegmatis) differently in the absence of Lsr2. Toward this end, we performed a transcriptional analysis of WT and Δ*lsr2* M. smegmatis strains growing under hypoxia. A modified rapid anaerobic dormancy (RAD) model was used to induce oxygen deficiency conditions (see Materials and Methods). We observed significant growth inhibition of the Δ*lsr2* M. smegmatis strain under hypoxia compared to that under a normal atmospheric oxygen concentration (Fig. S1A and B).

**FIG 4:**
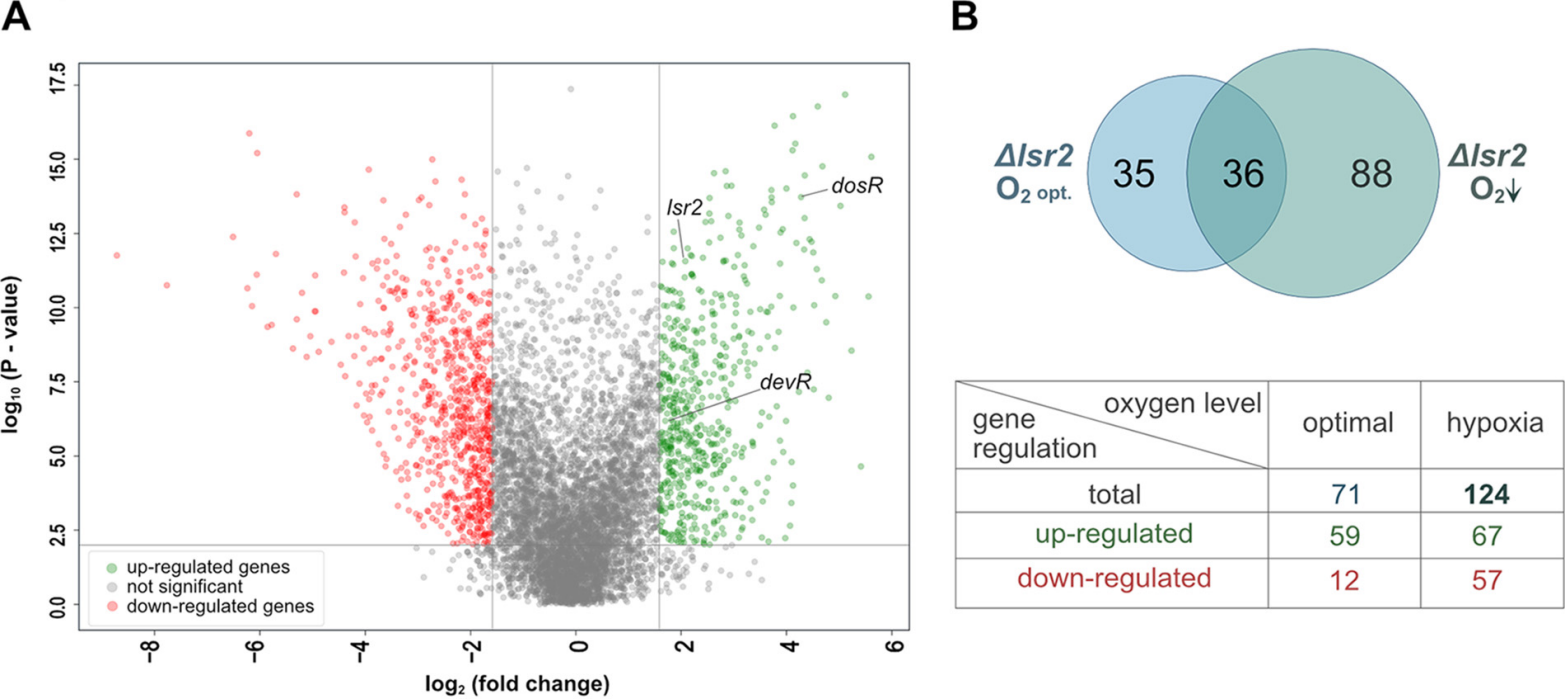
Analysis of the M. smegmatis regulon under hypoxia. (A) Volcano plot of RNA-seq transcriptome data displaying the pattern of gene expression values for the WT M. smegmatis strain (mc^2^ 155) under oxygen depletion. (B) Diagram comparing sets of genes regulated by Lsr2 under optimal oxygen (O_2 opt._) and hypoxic (O_2_↓) conditions. The table contains general information regarding the Lsr2 regulons observed under optimal O_2_ and hypoxia.

First, we analyzed changes in global gene expression in the WT strain (mc^2^ 155) under hypoxia (Fig. 4A). Our RNA-seq analysis revealed that there was a broad transcriptional response to hypoxic stress, with altered transcription seen for 1,367 genes (578 and 789 were increased and decreased, respectively), constituting nearly 20% of the M. smegmatis open reading frames (ORFs) (Fig. 4A). Similarly to Martini et al. (26), we observed significant increases in the transcript levels of *dosR* (MSMEG_3944) and *devR* (MSMEG_5244), whose gene products are known to mediate the response to hypoxia in M. tuberculosis. This verified that the RAD model had been applied correctly and was relevant in this system. Among the downregulated genes, 38 encoded ribosomal proteins (rp), most of which are organized in operons (Fig. S6). Our RNA-seq data demonstrated that constricted access to oxygen triggers the expression of genes encoding various regulatory proteins, such as transcription factors (14 genes), sigma factors (3 genes), and serine-threonine kinases (2 genes) (Table S2). The transcript expression of *lsr2* increased under hypoxia (log_2_ fold change = 1.65), prompting us to determine the level of Lsr2 in this system. Our RT-qPCR analysis confirmed the RNA-seq data by showing that the level of *lsr2* mRNA was 4.2 times higher under hypoxia (Fig. S5), and our Western blot analysis showed that the Lsr2 level was increased under hypoxia (Fig. S1C). We hypothesized that the upregulation of Lsr2 might affect the composition (i.e., gene number and/or content) of its regulon. Indeed, we found that more genes (124 genes) were controlled by Lsr2 under hypoxia than under the optimal oxygen condition (71 genes) (Fig. 4B; Table S2). Under the optimal oxygen condition, Lsr2 mainly downregulates genes; under hypoxia, in contrast, it could both down- and upregulate genes (the expression of 67 and 57 genes was increased and decreased, respectively, in the Δ*lsr2* mutant under hypoxia) (Fig. 2B). The levels of expression of only 36 of the Lsr2-dependent genes were comparable under optimal oxygen and hypoxic conditions. Thus, we speculate that the oxygen level affects not only the level of Lsr2 but presumably also its activity and/or binding mode. We additionally observed that under hypoxia, the expression levels of some rp genes (19 rp genes) in Δ*lsr2* cells were even lower than in WT cells (Fig. S5). This appears to at least partially explain the poor growth of the Δ*lsr2* strain under hypoxia (Fig. S1B).

10.1128/mSphere.00290-21.6 FIG S6 Gene expression analysis. Heat map illustrating RNA-seq differential expression data for genes encoding the ribosomal proteins (rp). (A) M. smegmatis mc^2^155 wild-type (WT_hyp_) and *Δlsr2* (*Δlsr2*_hyp_) rp gene expression profiles with oxygen depletion in reference to that of the wild type under optimal conditions. (B) M. smegmatis *Δlsr2* (*Δlsr2*_hyp_) rp gene expression profile with oxygen depletion in reference to that of the wild type under the same conditions. Download FIG S6, TIF file, 0.4 MB. Copyright © 2021 Kołodziej et al. 2021 Kołodziej et al. https://creativecommons.org/licenses/by/4.0/ This content is distributed under the terms of the Creative Commons Attribution 4.0 International license.

We also investigated whether MSMEG_1060 takes part in the cellular response to hypoxia. For this purpose, we constructed an M. smegmatis strain with deletion of the *msmeg_1060* gene and analyzed its growth rate under oxygen depletion. In contrast to what occurred after deletion of *lsr2*, deletion of *msmeg_1060* had no observable effect on M. smegmatis growth under oxygen depletion (Fig. S1B). However, RT-qPCR analysis revealed that the transcript level of *msmeg_1060* was profoundly decreased (approximately 6.5 times) under hypoxia, presumably due to the increased level of its repressor, Lsr2 (Fig. S5).

Together, these results show that Lsr2, but not MSMEG_1060, mediates the adaptation of M. smegmatis to oxygen depletion and that Lsr2 is a global transcription regulator that crucially modulates genes involved in adaptation to changing oxygen levels.

### The lack of MSMEG_1060 or Lsr2 increases cell susceptibility to antibiotics.

**FIG 5:**
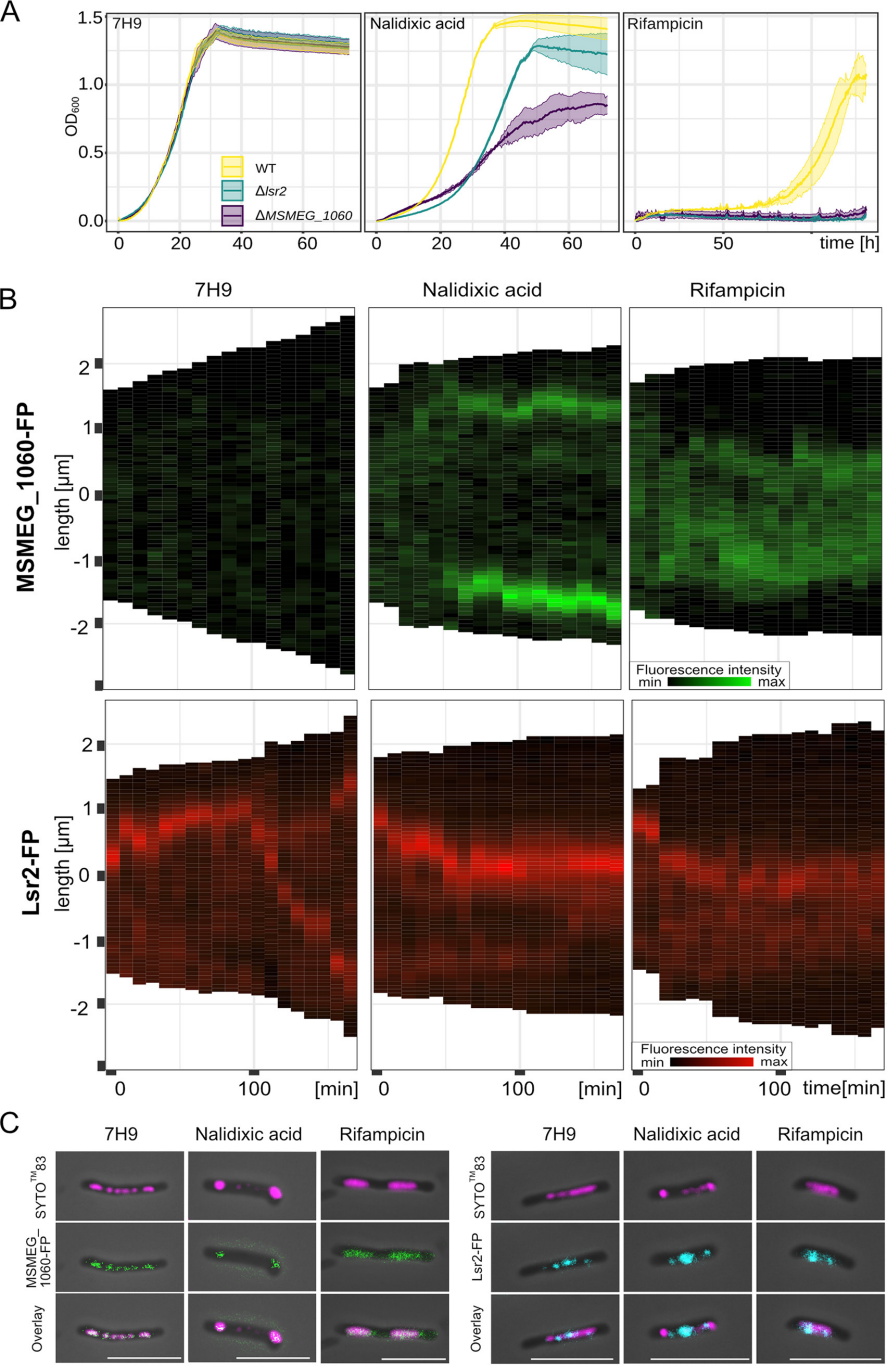
Real-time localization of MSMEG_1060-mTurquoise2 and Lsr2-FP fusion proteins in M. smegmatis cells under optimal growth conditions and after exposure to nalidixic acid or rifampicin. (A) Growth curves of the M. smegmatis WT, Δ*msmeg_1060*, and Δ*lsr2* strains under optimal growth conditions (7H9 medium, left panel) and during exposure to nalidixic acid (50 mg/liter) or rifampicin (5 mg/liter). (B) Kymograph presenting MSMEG_1060-mTurquoise2/Lsr2-mCherry fluorescence over time under optimal conditions and during antibiotic treatment. (C) Microphotographs showing representative MSMEG_1060-mTurquoise2 and Lsr2-FP cells stained with the nucleic acid dye SYTO 83 when grown under optimal conditions and after exposure for 200 min to nalidixic acid (50 mg/liter) or rifampicin (5 mg/liter).

A growing body of evidence suggests that NAP-mediated adaptation strategies help bacteria endure unfavorable conditions, including exposure to antibiotics (6, 32). Therefore, we sought to examine if MSMEG_1060 and Lsr2 proteins help M. smegmatis survive in the presence of antibiotics. For that purpose, we used nalidixic acid (Ndx) (33) and rifampicin (34), which inhibit DNA gyrase and RNA polymerase, respectively. Sublethal concentrations of the antibiotics were applied, with the goal of triggering subtle and observable antibiotic-induced changes without rapidly killing the mycobacterial cells (see Materials and Methods and reference 33). Analysis of growth curves (for details, see Materials and Methods) revealed that strains lacking MSMEG_1060 or Lsr2 were more susceptible to nalidixic acid and rifampicin than the WT (Fig. 5A). To further investigate this phenomenon, we used fluorescent reporter strains (MSMEG_1060-mTurquoise2 and Lsr2-mCherry) and a microfluidic CellASIC ONIX platform to perform real-time monitoring of the protein dynamics during antibiotic treatment (33, 35). Surprisingly, we noted pronounced changes in the subcellular localization of MSMEG_1060 during antibiotic treatment (Fig. 5B and C); before treatment, MSMEG_1060-mTurquoise2 was visualized as foci that were evenly distributed along the nucleoid (Fig. 3C), but soon after the addition of nalidixic acid, the fusion protein was visible as two discrete bright foci located at opposite poles of the cell (Fig. 5B and C). Notably, we recently demonstrated that Ndx exposure causes the M. smegmatis nucleoid to substantially shrink while preserving its structure and integrity (33). Thus, we speculated that MSMEG_1060 nucleoprotein complexes may protect chromosomal DNA against DNA fragmentation. To test this hypothesis, we examined the localization of MSMEG_1060-mTurquoise2 in relation to the SYTO 83-stained nucleoid after cells were exposed to nalidixic acid. We observed that MSMEG_1060-mTurquoise2 colocalized with the nucleoid following exposure to nalidixic acid (Fig. 5C). The MSMEG_1060-mTurquoise2 foci also shrank under rifampicin exposure, but in this case, they occupied the central part of the cell. Staining with SYTO 83 showed that the two fluorescence signals (i.e., mTurquoise2 and SYTO 83) also overlapped in this case. Moreover, the fluorescence signal of MSMEG_1060-mTurquoise2 seemed more intensive after antibiotic treatment. There was no significant change in the transcript expression level of MSMEG_1060 after antibiotics exposure, as indicated by RT-qPCR (Text S1; Fig. S5). Thus, the higher intensity of the MSMEG_1060-mTurquoise2 fluorescence signal after antibiotic treatment appears to result from the accumulation of MSMEG_1060 foci at the shrunken nucleoid.

In contrast to MSMEG_1060-FP (FP, fluorescent protein), Lsr2-FP does not occupy the entire nucleoid but forms one large nucleoprotein complex that duplicates during the course of DNA replication (23) (Fig. 5B). Unlike with our findings for MSMEG_1060-FP, we observed that treatment with nalidixic acid or rifampicin did not substantially alter the localization of Lsr2-FP, which remained in the middle of the cell, irrespective of the antibiotic used (Fig. 5B and C).

**FIG 6:**
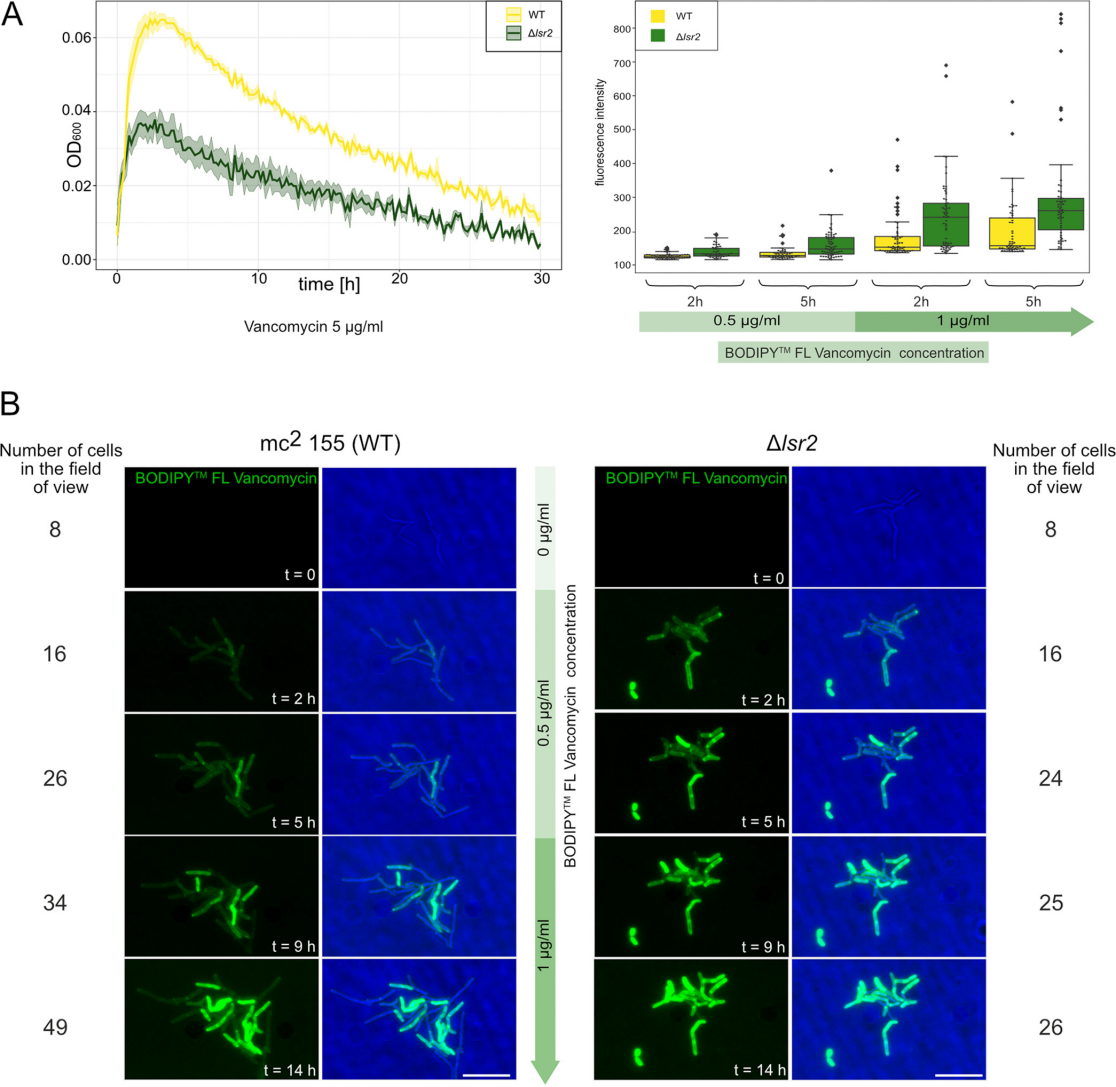
Comparison of vancomycin susceptibilities and levels of incorporation of vancomycin-BODIPY into the cell wall of WT and Δ*lsr2* cells. (A) Growth curves of the various M. smegmatis strains in the presence of 5 μg/ml vancomycin (left panel). The fluorescence intensity of vancomycin-BODIPY incorporated into the cell wall was measured at four different time points (right panel). Maximal fluorescence intensity was observed for the lateral cell wall away from the division site (*n* = 50 to 67 cells for each time point). (B) Time-lapse microfluidic microscopy (TLMM) analysis of vancomycin-BODIPY incorporation. Cells were exposed to 0.5 μg/ml vancomycin-BODIPY for 7 h, whereupon the concentration was increased to 1 μg/ml for the next 7 h. The number of surviving cells is shown alongside the micrographs. Bar, 5 μm.

Since the lack of Lsr2 altered the cell envelope properties, we speculated that the increased susceptibility of the Δ*lsr2* strain may be at least partially explained by the increased ability of the applied drugs to penetrate into the cells and not by the absence of Lsr2 *per se*. We also hypothesized that changes in the cell envelope, which were mirrored by changes in the morphology and smoothness of Δ*lsr2* cells, may have directly altered the susceptibility to antibiotics that inhibit cell wall synthesis. To test these hypotheses, we used green fluorescent protein (GFP)-labeled vancomycin (vancomycin-BODIPY; BODIPY FL vancomycin). Our growth curve analysis showed that the Δ*lsr2* strain was more susceptible to vancomycin than the WT strain (Fig. 6A, left panel). Time-lapse fluorescence microscopy (TLFM) experiments revealed that vancomycin-BODIPY was incorporated faster into the cell walls of Δ*lsr2* cells than of WT cells. The fluorescence intensity measured at four different time points during incubation with vancomycin-BODIPY was higher in the Δ*lsr2* strain than in the WT strain (Fig. 6B, right panel).

In summary, our data collectively suggest that the novel NAP MSMEG_1060 and the known NAP Lsr2 appear to mediate different adaptation strategies that help mycobacterial cells cope with exposure to antibiotics.

## DISCUSSION

Mycobacteria possess a unique set of NAPs, which are involved in various cellular processes. M. smegmatis is a saprophyte whose genome encodes significantly more genes (M. smegmatis mc^2^, 6,938 genes) than that of M. tuberculosis (M. tuberculosis H37Rv, 4,173 genes) (https://mycobrowser.epfl.ch/). Unlike M. tuberculosis, M. smegmatis possesses a gene called *MSMEG_1060*, which encodes an Lsr2-like protein. Here, we show that Lsr2 contributes to repressing dozens of genes, including *MSMEG_1060*, and that both Lsr2 and MSMEG_1060 are involved in the ability of M. smegmatis to adapt to changing environmental conditions.

### Lsr2 and MSMEG_1060 are paralogues with different binding specificities and chromosomal localizations.

Paralogues of Lsr2 have been identified in *Streptomyces* (e.g., in Streptomyces coelicolor and Streptomyces venezuelae) (13) and recently in M. smegmatis (10), but none has been thoroughly characterized to date. Our work (Fig. 3A) and previous *in vitro* analyses (17, 18, 36) revealed that, like H-NS, Lsr2 preferentially binds AT-rich DNA fragments. Our ChIP-seq analysis confirmed that Lsr2 prefers AT-rich regions (Fig. 1), but as with the situation seen in *S. venezuelae* (13), our ChIP-seq data did not allow us to define the consensus sequence for the Lsr2 binding motif.

Unlike Lsr2, MSMEG_1060 binds GC-rich fragments, albeit with lower affinity than it shows for DNA fragments with higher AT contents (Fig. 3A). Sequence comparison revealed that the MSMEG_1060 and Lsr2 proteins had moderate sequence identity (34%), particularly within the C-terminal domain responsible for DNA binding (Fig. S4E). MSMEG_1060 does not contain the Q/RGR motif that is typical of Lsr2 and H-NS. The motif belongs to a short AT hook-like loop that selectively interacts with the DNA minor groove and exhibits its highest affinity for AT-rich sequences (17, 18). Previous work demonstrated that mutations in the Q/RGR motif abolished the DNA binding activity of Lsr2 and H-NS (17, 18). Thus, the relatively low homology within the DNA binding domains between MSMEG_1060 and Lsr2 proteins and the lack of the characteristic Q/RGR motif in MSMEG_1060 may explain to some extent the differences in their DNA binding specificities. Interestingly, MSMEG_1060 has a tail (20 amino acids) that is rich in basic amino acids and is not found in Lsr2 (Fig. S4E). We hypothesize that this basic tail may increase the binding affinity of MSMEG_1060 or the stability of MSMEG_1060-DNA complexes. Our hypothesis is consistent with *in vitro* findings indicating that MSMEG_1060 exhibits a higher affinity toward DNA fragments than does Lsr2 (EMSAs) (Fig. 3A). In contrast to the DNA binding domains, the N termini of MSMEG_1060 and Lsr2 exhibit some sequence homology (Fig. S4E), potentially suggesting that these proteins may form hetero-oligomers.

As MSMEG_1060 and Lsr2 display different DNA binding properties *in vitro*, we speculated that their subcellular localizations might differ. Indeed, we found that MSMEG-1060 is distributed uniformly along the entire nucleoid (Fig. 3B and 5C), while Lsr2 forms a highly dynamic nucleoprotein complex (or two complexes), as we recently demonstrated (Fig. 5C) (23). Thus, the subcellular localizations of MSMEG_1060 and Lsr2 reflect their DNA binding preferences.

Since we found that Lsr2 represses *MSMEG_1060* gene expression, we cannot exclude the possibility that there is a regulatory interplay between MSMEG_1060 and Lsr2. Further studies are needed to investigate the reciprocal interaction(s) between these proteins.

### Lsr2 is a global gene regulator that targets AT-rich DNA sequences.

Previous work on Lsr2 from M. tuberculosis showed that, similarly to other H-NS-like proteins, Lsr2 acts as a repressor of gene expression (19). In this study, we combined RNA-seq and ChIP-seq data to elucidate a regulatory mode for Lsr2-mediated gene expression.

Our ChIP-seq analysis identified nearly 600 Lsr2 binding sites that are clustered within the AT-rich regions of the M. smegmatis chromosome (Fig. 1A). Notably, these regions frequently colocalized with *in silico*-predicted genomic islands (Fig. 1A). Thus, as in M. tuberculosis (17, 18, 37, 38), Lsr2 from M. smegmatis might be involved in regulating genes from horizontally transferred genomic islands and other AT-rich regions.

Our integrative analysis of RNA-seq and ChIP-seq data revealed that Lsr2 directly represses less than half of the genes in its regulon, including its paralogue *MSMEG_1060*; 28 of the putative promoters of these downregulated genes contained Lsr2 binding sites. Moreover, the presence of a relatively high and evenly distributed AT content within the Lsr2-dependent promoters (Fig. 1C) presumably favors the binding and spreading of multiple Lsr2 molecules along with the DNA. Our ChIP-seq data support this hypothesis; the relatively high average fragment length (405 bp) and the occurrence of extremely long reads (4% of reads are 1,000 bp or longer) suggest that Lsr2 tends to spread across the DNA strand (Fig. S2B). Thus, like other members of the H-NS protein family, the DNA binding and oligomerization of Lsr2 along an AT-rich promoter presumably leads to DNA stiffening and eventually to efficient gene silencing.

Notably, the majority of the Lsr2 binding sites identified by our ChIP-seq analysis (532 sites; 91%) are not directly (or at all) associated with transcriptional regulation. These sites may play an architectural role in the formation of massive nucleoprotein Lsr2-DNA complexes that presumably contribute to maintaining the proper organization of the newly synthesized DNA (23). However, we cannot exclude the possibility that Lsr2-induced changes in DNA topology and/or DNA accessibility may indirectly affect the expression levels of some genes, particularly under unfavorable conditions (e.g., oxygen depletion [see below]). Previous studies suggested that Lsr2 may oligomerize (via its N-terminal domain) and form bridged nucleoprotein complexes that connect distant DNA binding sites (13, 15). Thus, the trapping of genes within a DNA loop may be an additional mode of gene regulation employed by Lsr2. Our ChIP-seq data do not exclude this hypothesis; the average distance between the two closest binding sites was calculated to be around 6,000 bp (ranging from 1,933 to 15,390 bp) (Fig. S2B), suggesting that at least a few genes may be trapped by Lsr2. Our combined RNA-seq and ChIP-seq analyses revealed a set of nine genes (*MSMEG_4727* to *MSMEG_4735*) flanked by the Lsr2 binding sites that are repressed either directly by binding promoter regions or indirectly by a DNA loop that bridges these binding sites by means of Lsr2 (Fig. 2A and B). Transcriptional analysis performed by Martini et al. (26) does not rule out any of these possibilities; these nine genes are not organized into an operon. Our EMSA confirmed that the upstream region of the *MSMEG_4727* gene (involved in LOS synthesis) is bound by Lsr2 (see Fig. S4C and D). Moreover, TLC analysis of crude lipid fractions showed that, unlike the WT strain, the Δ*lsr2* strain produces a detectable amount of LOSs (Fig. 2C).

Further analyses, including the application of high-throughput, next-generation chromosome conformation capture (3C) technologies (Hi-C) are needed to shed additional light on how Lsr2 impacts the local chromosome structure, including whether DNA loop formation may regulate the expression of genes trapped by Lsr2.

### Lsr2 and MSMEG_1060 help M. smegmatis cells cope with stress.

M. smegmatis, as an obligate aerobic saprophyte, has a lifestyle that is markedly different from that of the intracellular pathogen M. tuberculosis. To grow and survive, the free-living soil bacterium must quickly adapt to changing environmental conditions, including exposure to various stresses. Among the many possible adaptation strategies, NAP-induced changes in chromosome organization resulting in physical protection of the DNA and/or altered gene expression appear to be the most rapid adaptation strategies (6, 19, 32) The Δ*lsr2* M. smegmatis strain exhibits a number of morphological disorders that are related to changes in the outer membrane composition. Cells devoid of Lsr2 produce more LOSs (Fig. 2), which contain a hydrophilic carbohydrate moiety and are found in the outer membrane of some mycobacterial species (39). In M. kansasii, LOSs have also been associated with colony smoothness, sliding motility, and the inability to form a biofilm. Interestingly, unlike the smooth variant of M. kansasii, the rough, LOS-deficient variants of M. kansasii are capable of causing chronic systemic infection in mice (40, 41). Recent studies have suggested that, during its evolution, M. tuberculosis became more hydrophobic by deletion of genes encoding proteins that are involved in the synthesis of polar lipids, including LOSs and mas synthase. It is believed that such a change could have enhanced the capability of aerosol transmission, affecting virulence and pathogenicity (32). In the free-living saprophyte M. smegmatis, we presume that the penetration (via sliding motility) and colonization (via biofilm formation) of a terrestrial environment might be facilitated by modulation of the level and/or activity of Lsr2 (e.g., by the kinase PknB) (42).

Lsr2 is required for the ability of M. tuberculosis to adapt to changing oxygen levels, including hypoxia (19). We also observed that the M. smegmatis strain lacking Lsr2 is more susceptible to a decreased oxygen level (Fig. S1). Unlike with M. tuberculosis (19), our analysis of M. smegmatis revealed that more genes were regulated (directly or indirectly) by Lsr2 under oxygen depletion than under an optimal oxygen concentration (Fig. 4). We expect that this change in the Lsr2 regulon arises via an increase in the level of Lsr2 under hypoxia. Under the optimal condition, Lsr2 acts mainly as a downregulator, as the transcription of only 17% of the regulated genes was decreased in the Δ*lsr2* strain; under hypoxia, in contrast, Lsr2 activated 46% of its regulated genes (Fig. 4B). Interestingly, the genes that were activated under hypoxia included many encoding ribosomal proteins (rp). The transcription levels of rp genes were lower in WT cells under hypoxia than the levels seen under the optimal oxygen condition, and this tendency was even more pronounced in Δ*lsr2* cells (Fig. S6). This might explain the growth defect seen in *Δlsr2* cells grown under unfavorable oxygen conditions.

As a free-living organism, M. smegmatis is also exposed to other environmental stresses, such as antibiotics produced by various soil microorganisms (mainly *Streptomyces*) (32, 43, 44). We expected that both Lsr2 and its paralogue MSEMG_1060 would play important roles in the adaptation of M. smegmatis to unfavorable environmental conditions. Our initial experiments indicated that the lack of Lsr2 or MSMEG_1060 increased the susceptibility of M. smegmatis cells to antibiotics (Fig. 5A). Given that these paralogues exhibit different DNA binding specificities and chromosomal localizations (Fig. 3), we hypothesized that they would protect M. smegmatis cells from antibiotics in different ways. Previous studies demonstrated that in M. tuberculosis and M. smegmatis, Lsr2 is not required to protect DNA against damage from oxidative stress and other DNA-damaging agents (19). This is in line with the subcellular localization of Lsr2, which is not distributed throughout the nucleoid but rather forms one or two nucleoprotein complexes (Fig. 5C) (23). We speculate that changes in the LOS level, which were presumably mirrored by differences in the cell envelope properties of Δ*lsr2* cells, may have directly altered the penetration of antibiotics (45). LOS is an amphipathic molecule that consists of a hydrophobic lipid part that anchors the LOSs to the outer membrane and a hydrophilic carbohydrate part (27, 46) that might promote the penetration of hydrophilic antibiotics that inhibit cell wall synthesis (e.g., vancomycin). Indeed, the Δ*lsr2* strain was more susceptible to vancomycin than the WT strain (Fig. 6A) and fluorescently labeled vancomycin-BODIPY was more quickly incorporated into the cell wall of the Δ*lsr2* strain (Fig. 6).

We demonstrated that cells lacking MSMEG_1060 are more susceptible to nalidixic acid, a DNA gyrase inhibitor that induces pronounced changes in DNA integrity (33, 47). In contrast to Lsr2, MSMEG_1060 showed dispersed distribution across the nucleoid and thus, as with other nonspecific DNA binding NAPs, including the E. coli H-NS paralogue StpA (48, 49), may protect DNA by coating it. Further analyses using various antibiotics, particularly those that target the DNA, are needed to support our hypothesis.

In sum, our studies provide novel insights into the role of Lsr2 paralogues in M. smegmatis. This saprophytic Mycobacterium is frequently used as a valuable model organism to study the biology of tubercle bacilli. However, M. smegmatis has a lifestyle distinct from that of M. tuberculosis. Here, we demonstrate that Lsr2 and MSMEG_1060 exhibit differences in their DNA binding modes, but both contribute to the ability of M. smegmatis cells to adapt to a changing environment. MSMEG_1060, as a paralogue of Lsr2, presumably acquired its novel functions during the evolution of saprophytic mycobacteria. In the future, a comprehensive analysis of MSMEG_1060 is needed to elucidate its detailed role in chromosomal organization and transcriptional regulation.

## MATERIALS AND METHODS

### DNA manipulations, bacterial strains, and culture conditions.

Bacterial strain construction and culture were performed as described previously (23). A detailed description of the bacterial culture conditions is provided in the Text S1 in the supplemental material. Briefly, plasmids used for M. smegmatis mc^2^ 155 transformation were reproduced in the E. coli DH5α strain. M. smegmatis strains were grown in 7H9 broth supplemented with 10% oleic acid-albumin-dextrose-catalase (OADC; BD) and 0.05% Tween 80. Enzymes and reagents were provided by Thermo Fisher, Roth, and Merck (Sigma-Aldrich). Oligonucleotides were synthesized by Merck (Sigma-Aldrich), and sequencing was performed by Microsynth. The rapid anaerobic dormancy (RAD) model (42) was used to shift M. smegmatis cultures from aerobic growth to hypoxia.

The construction of the M. smegmatis mc^2^ 155 mutant strains is described in Text S1. The utilized bacterial strains, plasmids, and oligonucleotides are listed in Tables S3 in Text S1.

### RNA-seq.

For total RNA sequencing (RNA-seq), RNA was isolated as described in Text S1. Illumina system-compatible sequencing libraries were prepared essentially as described previously (50). RNA quantity and integrity were assessed using an Agilent 2100 Bioanalyzer (Agilent RNA 6000 Nano kit). Briefly, RNA samples were initially purified using magnetic beads (AMPure XP magnetic beads; Becton, Dickinson), rRNA was removed (Ribo-Zero rRNA removal kit; Illumina), and sequencing libraries were generated (KAPA stranded RNA-Seq kit; KAPA Biosystems) according to the manufacturer’s protocol. Illumina system-compatible adapters containing sample-specific 8-nucleotide-long barcoding sequences were ligated, and the cDNA libraries were PCR amplified. The resulting sequencing libraries were evaluated using an Agilent 2100 Bioanalyzer with a DNA 1000 chip and quantified by real-time PCR using the NEBNext Library Quant kit for Illumina (New England Biolabs). Raw sequencing data were generated on a NextSeq500 platform (Illumina) using paired-end 75-cycle sequencing run-compatible reagents (150 cycles, NextSeq 500/550 Mid Output v2 sequencing kit; Illumina). Libraries were prepared and sequenced in biological triplicates for each tested growth condition.

### Bioinformatics data analysis.

For RNA-seq analysis, the raw total RNA-sequencing data were subjected to initial demultiplexing and processed to remove library adapters (Cutadapt v.1.3 [51]). Short (<20 bp) and low-quality (<30%) reads were also filtered and removed (Sickle script v.1.33 [52]). The remaining high-quality reads were mapped to the M. smegmatis mc^2^ 155 genome (retrieved from the NCBI database, accession number NC_008596) using the Bowtie2 short-read aligner (53). The resulting BAM files, which contained read-mapping coordinates, were indexed and sorted with SAMtools v.1.7 (54) to allow data visualization (Integrative Genomics Viewer [55]) or counting of reads to gene features (HTSeq.count script [56]). The read counts for individual samples were merged into a count matrix, which was submitted to the online differential expression RNA-seq analysis platform Degust (http://doi.org/10.5281/zenodo.3501067). Default parameters were used for the analysis, and the voom/limma method (57) was chosen for final data evaluation. Differential expression data were visualized in the form of a volcano plot and heatmaps, using the Python Seaborn software suite and required dependencies (https://pypi.org/project/seaborn).

### ChIP-seq.

ChIP experiments were performed as described previously (7). Briefly, M. smegmatis strains producing Lsr2-FLAG_3_ and WT Lsr2 (negative control) were grown to log phase (optical density at 600 nm [OD_600_], 0.6) in liquid medium. The cells were fixed with 0.01% formaldehyde for 30 min, quenched with glycine, and washed three times with cold phosphate-buffered saline (PBS). To prepare lysates, pellets were disintegrated with silica beads and sonicated. The obtained lysates were centrifuged (5 min, 12,000 rpm, 4°C) and frozen. For immunoprecipitation, 700 μg of total protein was incubated on a rotary shaker for 4 h at 4°C with a 20-μl packed-gel volume of anti-FLAG M2 magnetic beads (Sigma-Aldrich) and then washed. Samples were processed in a final volume of 0.5 ml in two biological replicates, with input DNA controls (70 μg of total protein alone) included for each replicate. Immunoprecipitated samples were de-cross-linked and then digested with proteinase K (final concentration, 0.05 mg/ml). DNA was extracted using phenol-chloroform-isoamyl alcohol (25:24:1, vol/vol/vol) and precipitated with absolute ethanol. Library construction, Illumina sequencing, and analysis of ChIP-seq data were performed as described previously (7).

### Microscopy and cell staining.

For nucleoid acid staining, cells were incubated with SYTO 83 (Thermo Fisher; concentration, 0.5 μM) for 15 min, centrifuged (5,000 rpm/5 min), and plated on a microscope slide. Snapshots were taken immediately after sample preparation using a Leica DM6 B microscope with a 100× oil immersion objective. Real-time analyses were performed in liquid medium using a CellASIC ONIX platform and compatible B04A plates (Merck), as described previously (23, 33). In both cases, early-log-phase (OD_600_ ∼ 0.2 to 0.4) M. smegmatis cultures grown in liquid medium were used. For vancomycin-BODIPY (BODIPY FL vancomycin; Thermo Fisher) staining, cells loaded into the observation chamber were exposed to fresh 7H9-OADC-Tween 80 for 5 h, to 7H9-OADC-Tween 80 supplemented with 0.5 μg/ml vancomycin-BODIPY for 5 h, and then to 7H9-OADC-Tween 80 supplemented with 1 μg/ml vancomycin-BODIPY for 5 h (Fig. 3). All microfluidic experiments were performed under constant pressure (1.5 lb/in^2^). Images were recorded at 10-min intervals using a Delta Vision Elite inverted microscope equipped with a 100× oil immersion objective and an environmental chamber set to 37°C. Pictures were analyzed using the Fiji and R software packages (R Foundation for Statistical Computing, Austria; http://www.r-project.org), including the ggplot2 package (58).
