# Supplementary figures and images for: Lsr2 and Its Novel Paralogue Mediate the Adjustment of Mycobacterium smegmatis to Unfavorable Environmental Conditions
Source: mSphere. 2021 May 12;6(3):e00290-21. doi: 10.1128/mSphere.00290-21 (PMC8125055; doi:10.1128/mSphere.00290-21)

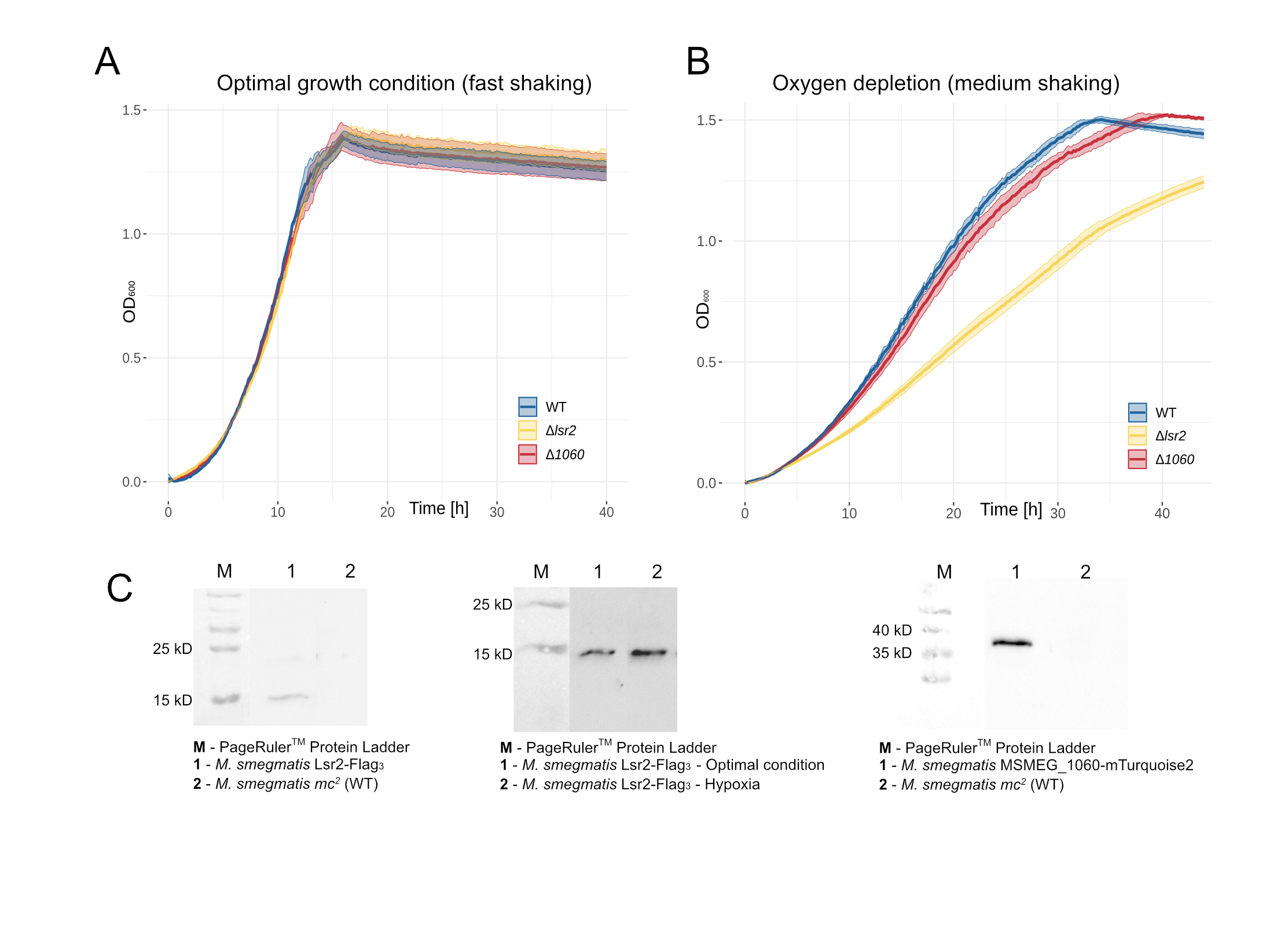

Supplement: FIG S1 [file mSphere.00290-21-sf001.tif]

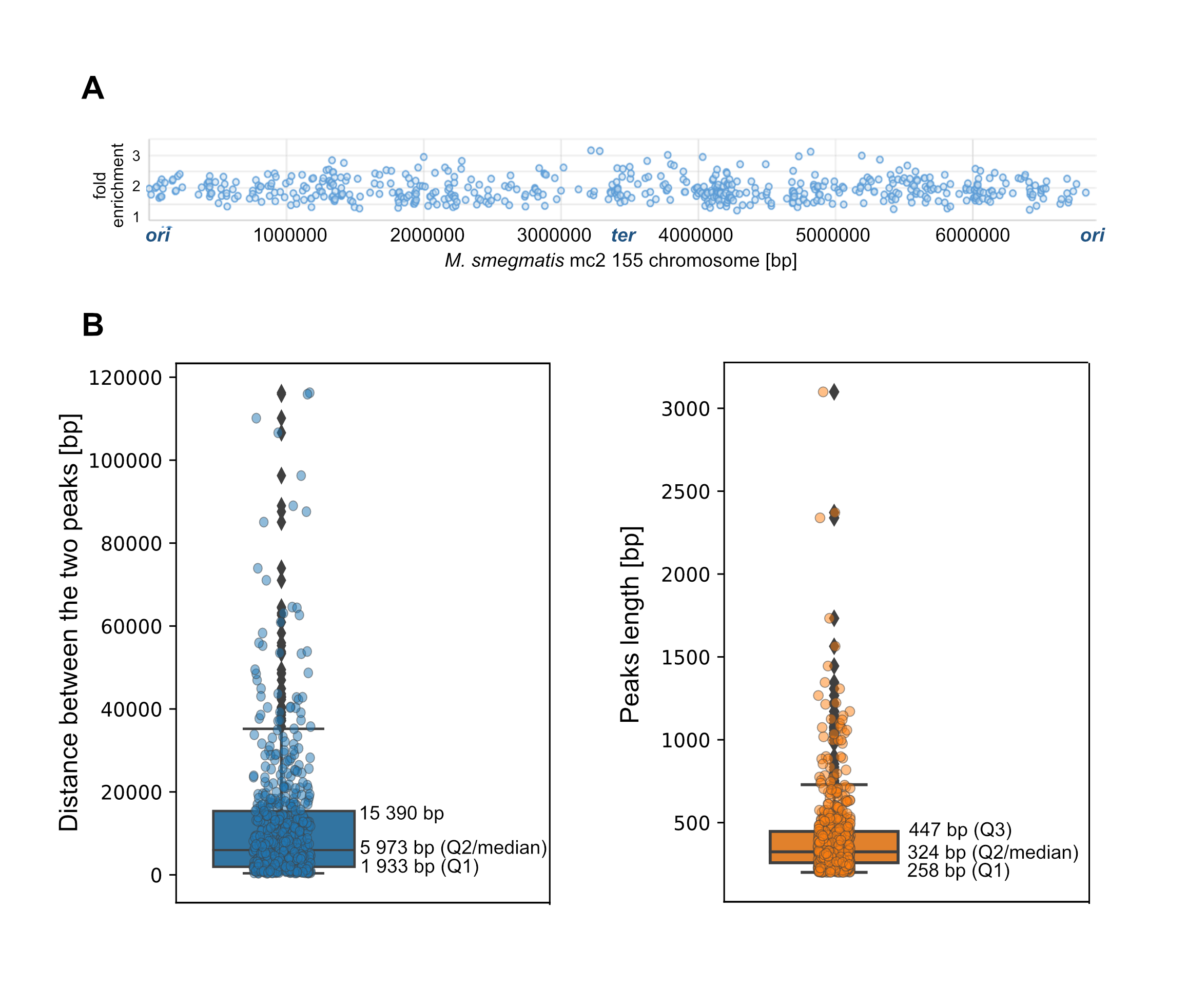

Supplement: FIG S2 [file mSphere.00290-21-sf002.tif]

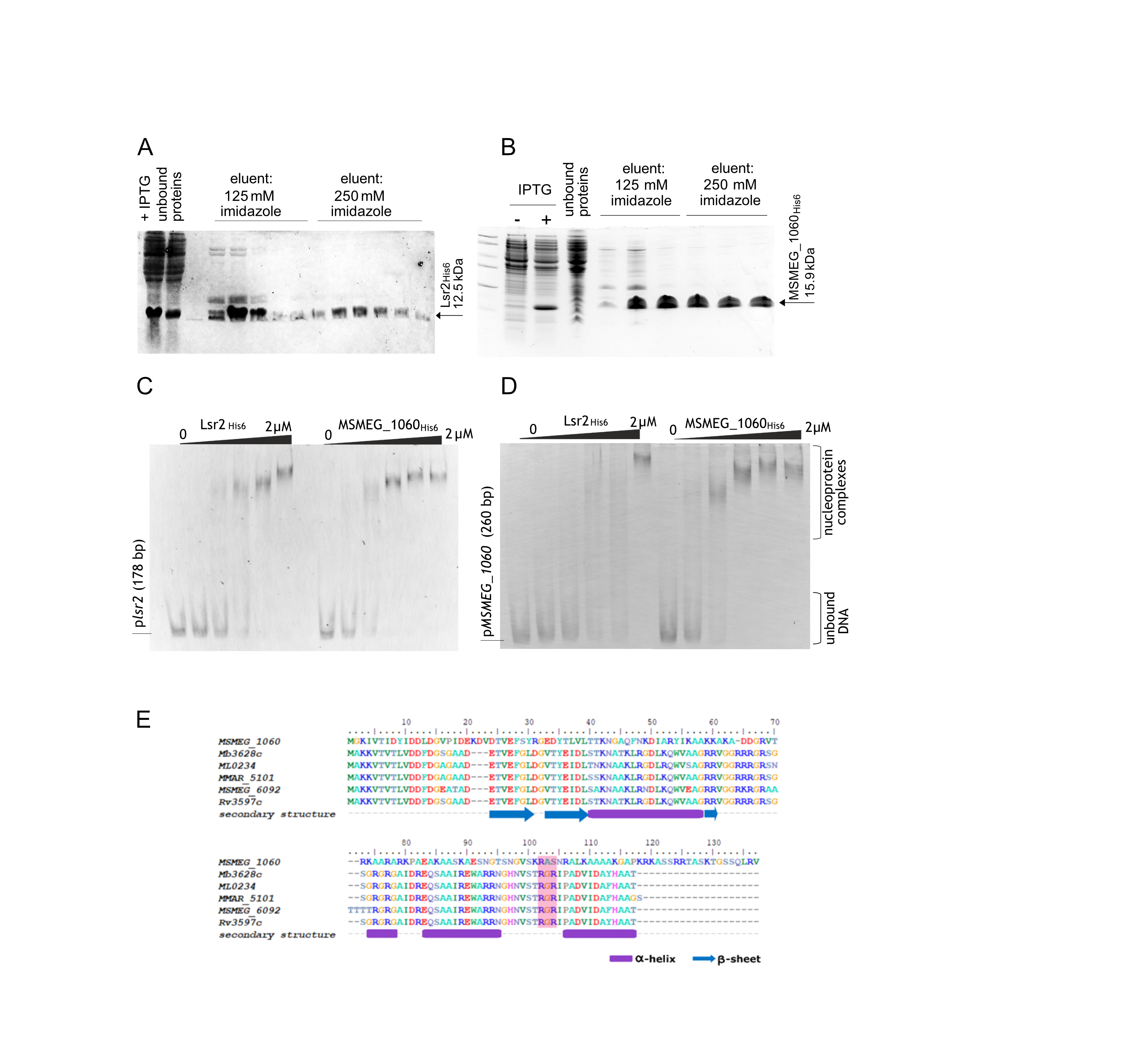

Supplement: FIG S4 [file mSphere.00290-21-sf004.tif]

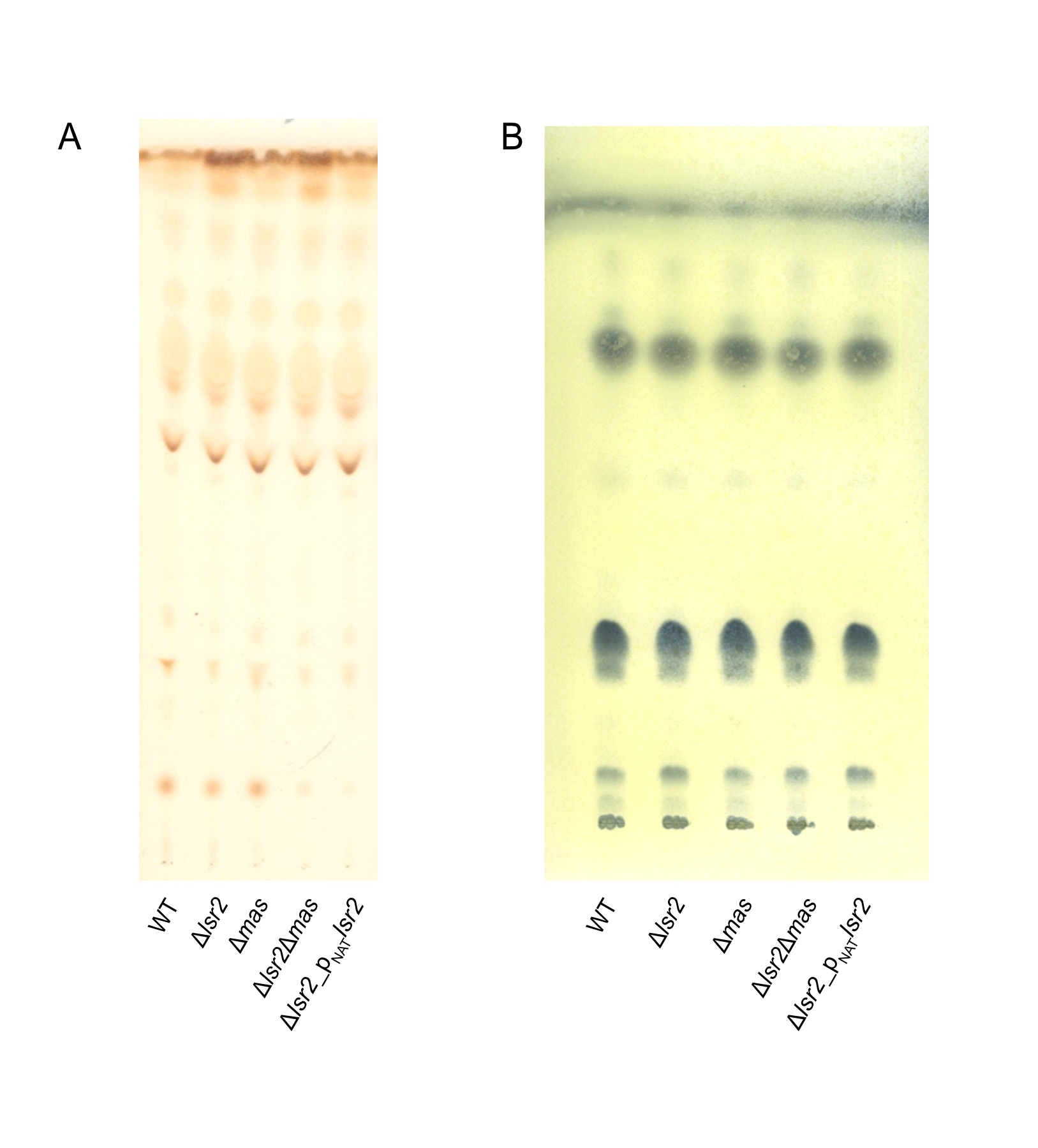

Supplement: FIG S3 [file mSphere.00290-21-sf003.tif]

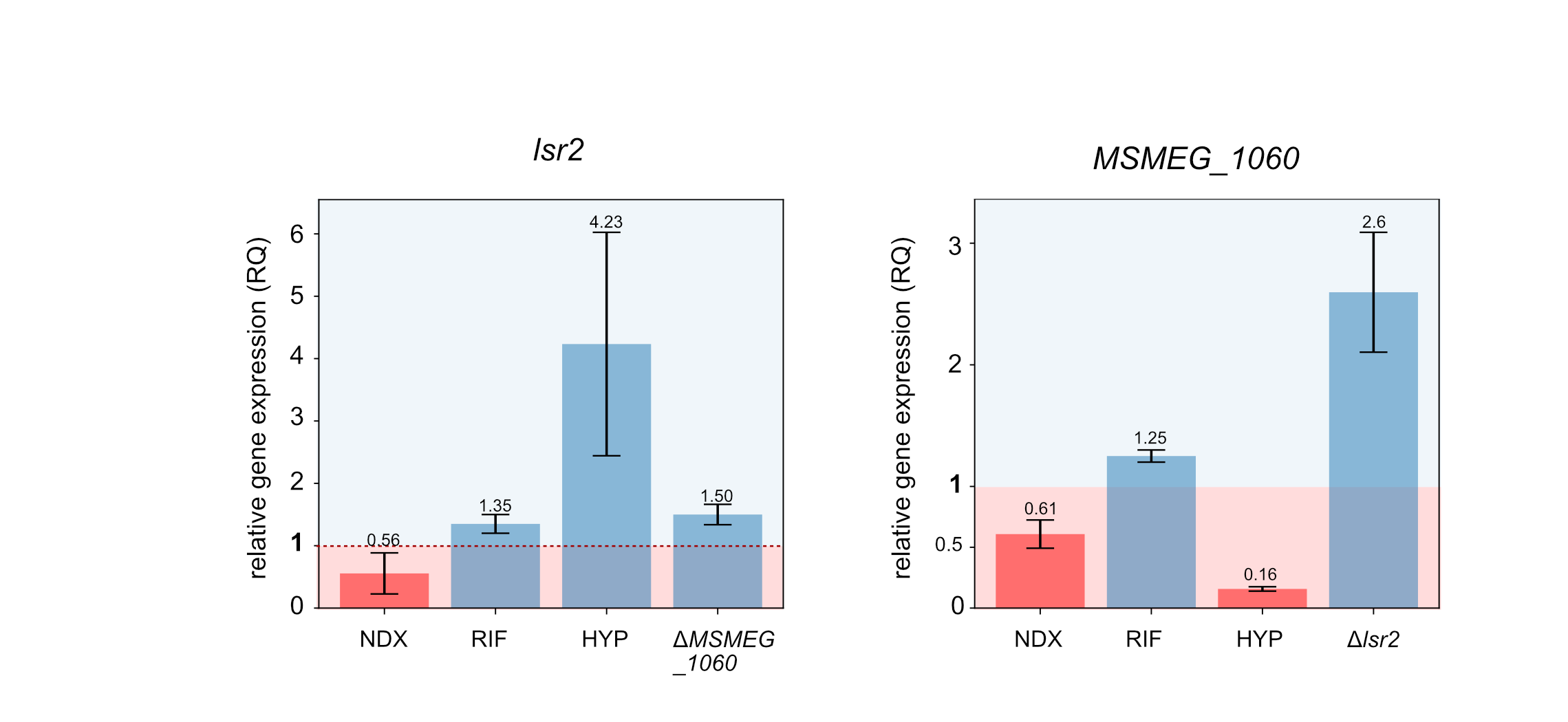

Supplement: FIG S5 [file mSphere.00290-21-sf005.tif]

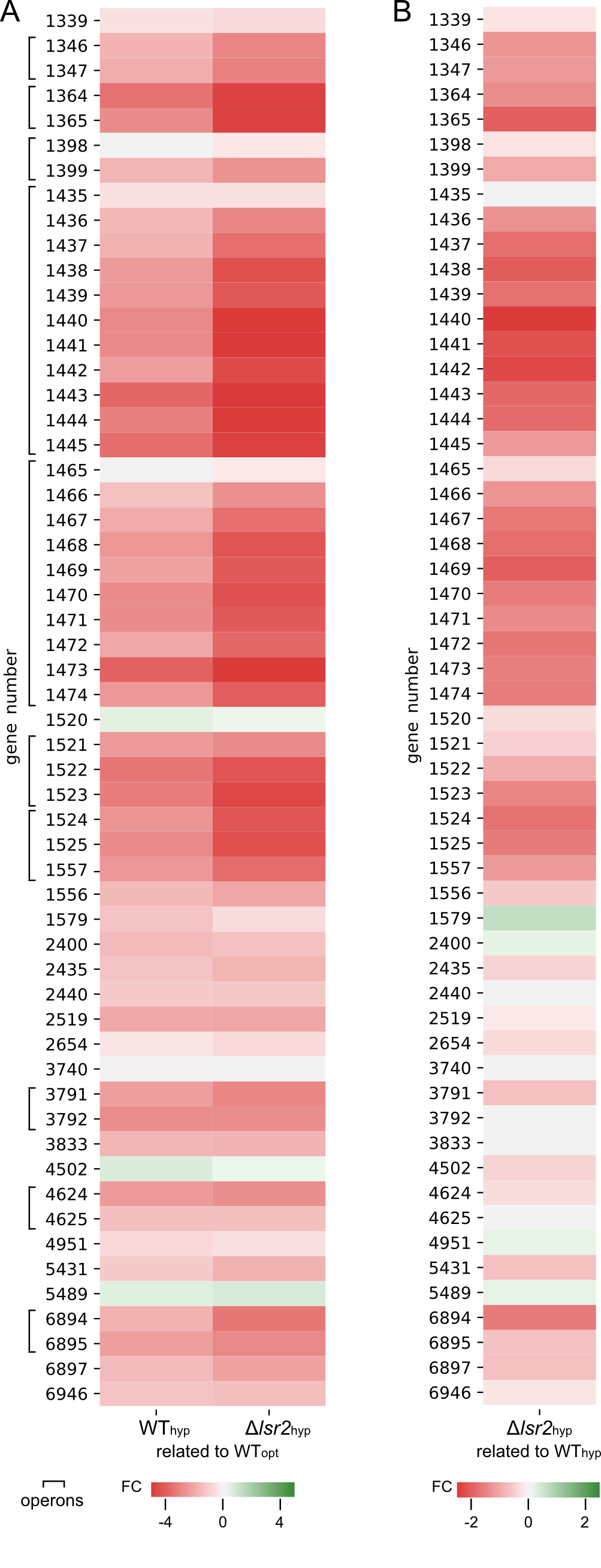

Supplement: FIG S6 [file mSphere.00290-21-sf006.tif]
