# Supplementary material for: Lsr2 and Its Novel Paralogue Mediate the Adjustment of Mycobacterium smegmatis to Unfavorable Environmental Conditions
Source: mSphere. 2021 May 12;6(3):e00290-21. doi: 10.1128/mSphere.00290-21 (PMC8125055; doi:10.1128/mSphere.00290-21)
Supplement: TABLE S1 [file mSphere.00290-21-st001.docx]

| **Table S1. Genes with altered expression in Δ*lsr2* strain compared to WT in optimal growth condition. Blue-marked genes are bounded by Lsr2 in the promoter region.** | | |
| --- | --- | --- |
| gene | **Annotation** | FC |
| **MSMEG_1128** | hypothetical protein | 5.0 |
| **MSMEG_1241** | hypothetical protein | 4.9 |
| **MSMEG_2827** | hypothetical protein | 4.6 |
| **MSMEG_5973** | hypothetical protein | 4.6 |
| **MSMEG_3727** | hypothetical protein | 4.1 |
| **MSMEG_3726** | alcohol dehydrogenase | 4.0 |
| **MSMEG_3720** | recombination activating protein 1 | 4.0 |
| **MSMEG_4730** | hypothetical protein | 4.0 |
| **MSMEG_3728** | hypothetical protein | 3.9 |
| **MSMEG_4729** | hypothetical protein | 3.8 |
| **MSMEG_1129** | D-amino-acid dehydrogenase | 3.8 |
| **MSMEG_5972** | hypothetical protein | 3.8 |
| **MSMEG_3719** | sodium/calcium exchanger protein | 3.7 |
| **MSMEG_4731** | acyl-CoA synthetase | 3.7 |
| **MSMEG_1240** | hypothetical protein | 3.7 |
| **MSMEG_3723** | pqqC; pyrroloquinoline quinone biosynthesis protein PqqC | 3.7 |
| **MSMEG_3724** | pqqB; pyrroloquinoline quinone biosynthesis protein PqqB | 3.6 |
| **MSMEG_3722** | bifunctional coenzyme PQQ synthesis protein C/D | 3.6 |
| **MSMEG_1239** | ISMsm4, transposase | 3.5 |
| **MSMEG_4728** | condensation domain-containing protein | 3.4 |
| **MSMEG_4732** | glycosyl transferase family protein | 3.3 |
| **MSMEG_4727** | mycocerosic acid synthase | 3.2 |
| **MSMEG_3725** | pqqA; coenzyme PQQ biosynthesis protein A | 3.2 |
| **MSMEG_3721** | pqqE; pyrroloquinoline quinone biosynthesis protein PqqE | 3.0 |
| **MSMEG_6147** | hypothetical protein | 3.0 |
| **MSMEG_3374** | hypothetical protein | 2.7 |
| **MSMEG_5958** | hypothetical protein | 2.5 |
| **MSMEG_5461** | hypothetical protein | 2.4 |
| **MSMEG_5460** | hypothetical protein | 2.4 |
| **MSMEG_5710** | hypothetical protein | 2.4 |
| **MSMEG_5501** | hypothetical protein | 2.4 |
| **MSMEG_5957** | algD; GDP-mannose 6-dehydrogenase | 2.3 |
| **MSMEG_3718** | hypothetical protein | 2.3 |
| **MSMEG_5231** | hypothetical protein | 2.3 |
| **MSMEG_5709** | hypothetical protein | 2.3 |
| **MSMEG_1238** | type III restriction enzyme, res subunit | 2.3 |
| **MSMEG_3578** | cyclase | 2.2 |
| **MSMEG_4733** | hypothetical protein | 2.2 |
| **MSMEG_1224** | rifampin ADP-ribosyl transferase | 2.2 |
| **MSMEG_1135** | hypothetical protein | 2.2 |
| **MSMEG_1253** | hypothetical protein | 2.2 |
| **MSMEG_3579** | transmembrane protein | 2.1 |
| **MSMEG_5971** | hypothetical protein | 2.0 |
| **MSMEG_5459** | hypothetical protein | 2.0 |
| **MSMEG_5708** | hypothetical protein | 2.0 |
| **MSMEG_1127** | transmembrane protein | 1.9 |
| **MSMEG_5711** | short chain dehydrogenase | 1.9 |
| **MSMEG_4859** | oxidoreductase, short chain dehydrogenase/reductase | 1.8 |
| **MSMEG_1247** | hypothetical protein | 1.8 |
| **MSMEG_5558** | hypothetical protein | 1.8 |
| **MSMEG_6579** | hypothetical protein | 1.8 |
| **MSMEG_2199** | hypothetical protein | 1.8 |
| **MSMEG_5438** | ksgA; dimethyladenosine transferase | 1.7 |
| **MSMEG_0200** | hypothetical protein | 1.7 |
| **MSMEG_2822** | pseudogene | 1.7 |
| **MSMEG_5230** | hypothetical protein | 1.7 |
| **MSMEG_1130** | hypothetical protein | 1.7 |
| **MSMEG_5092** | hypothetical protein | 1.6 |
| **MSMEG_4734** | hypothetical protein | 1.6 |
| **MSMEG_0242** | hypothetical protein | -1.6 |
| **MSMEG_1212** | hypothetical protein | -1.6 |
| **MSMEG_4640** | hypothetical protein | -1.6 |
| **MSMEG_5298** | hypothetical protein | -1.6 |
| **MSMEG_1599** | RNA polymerase sigma factor SigD | -1.7 |
| **MSMEG_1712** | ABC transporter periplasmic protein | -1.8 |
| **MSMEG_3446** | hypothetical protein | -1.8 |
| **MSMEG_3327** | hypothetical protein | -1.9 |
| **MSMEG_1859** | hypothetical protein | -2.1 |
| **MSMEG_5355** | hypothetical protein | -2.1 |
| **MSMEG_6093** | hypothetical protein | -2.1 |
| **MSMEG_4642** | hypothetical protein | -2.9 |
