# Supplementary material for: Lsr2 and Its Novel Paralogue Mediate the Adjustment of Mycobacterium smegmatis to Unfavorable Environmental Conditions
Source: mSphere. 2021 May 12;6(3):e00290-21. doi: 10.1128/mSphere.00290-21 (PMC8125055; doi:10.1128/mSphere.00290-21)
Supplement: TABLE S3 [file mSphere.00290-21-st003.docx]

| **Table S3. Strains, plasmids, and oligonucleotides used in this study** | | | | |
| --- | --- | --- | --- | --- |
| **Strains used in this study** | | | | |
| **Name** | **Relevant genotype** | | | **Source** |
| WT | *M. smegmatis* mc^2^ 155 | | | laboratory stock |
| Δ*lsr2* | *M. smegmatis* mc^2^ 155 Δ*lsr2* | | | (1) |
| Δ*lsr2*_p_NAT_*lsr2* | *M. smegmatis* mc2 155 ∆*lsr2 attBL5::pMV306p_NAT_lsr2* | | | (1) |
| Lsr2-Flag_3_ | *M. smegmatis* mc^2^ *lsr2::lsr2-flag_3_* | | | this study |
| Δ*MSMEG_1060* | *M. smegmatis* mc^2^ 155 Δ*MSMEG_1060* | | | this study |
| Δ*lsr2*Δ*MSMEG_1060* | *M. smegmatis* mc^2^ 155 Δ*lsr2*Δ*MSMEG_1060* | | | this study |
| Δ*mas* | *M. smegmatis* mc^2^ 155 Δ*MSMEG_4727* | | | this study |
| Δ*lsr2*Δ*mas* | *M. smegmatis* mc^2^ 155 Δ*lsr2*Δ*MSMEG_4727* | | | this study |
| Lsr2-mTurquoise2 | *M. smegmatis* mc^2^ *lsr2::lsr2-mTurquoise2* | | | (1) |
| MSMEG_1060-mTurquoise2 | *M. smegmatis* mc^2^ *MSMEG_1060::MSMEG_1060-mTurquoise2* | | | this study |
| **Plasmids used in this study** | | | | |
| **Name** | **Plasmid features** | | | **Source** |
| p2NIL | kanamycin resistance, *oriE*, suicide vector for allelic replacement | | | (2) |
| pGoal17 | ampicillin resistance, *oriE*, selective PacI cassette (Goal) with *lacZ*, *sacB* and *kanR* genes | | | (2) |
| p2NIL-MSMEG_1060-mTurquoise2-Goal | kanamycin resistance, *oriE*, *MSMEG_1060-* *mTurquoise2* fusion gene, PacI cassette | | | this study |
| p2NIL- Δ*MSMEG_1060-*Goal | kanamycin resistance, *oriE*, PacI cassette | | | this study |
| p2NIL- Δ*MSMEG_4727-*Goal | kanamycin resistance, *oriE*, PacI cassette | | | this study |
| p2NIL- *lsr2-flag_3_-* Goal | kanamycin resistance, *oriE*, PacI cassette | | | (1) |
| **Oligonucleotides used in this study** | | | | |
| **Name** | | **Sequence 5’ to 3’** | **General description** | |
| Ms_1061_Slic_Fv | | CGCCGACGACCTGCAGCTTCCTCCGATGTCG | MSMEG_1061 (flank_1) amplification to SLIC reaction (Δ*MSMEG_1060* strain) | |
| Ms_1061_Slic_Rv | | TTAGGTGACACTATAGAATACATAGGATCCATGCGCCAAGAGGTCACC |  |  |
| Ms_1059_Slic_Fv | | TAACTGTGATAAACTACCGCATTAAAGCTTTCACCGCCGAGGTTGACG | MSMEG_1059 (flank_2) amplification to SLIC reaction (Δ*MSMEG_1060* strain) | |
| Ms_1059_Slic_Rv | | CGGAGGAAGCTGCAGGTCGTCGGCGCG |  |  |
| d_mas_F1_**Hind**_Fw | | GTGATAAACTACCGCATTA**AAGCTT**GTGGTCGCACACACGGTCG | MSMEG_4726 and intergenic region (flank_1) amplification to SLIC reaction (Δ*mas* strain) | |
| d_mas_F1_Rv | | GGGTCAGGCCAGCAATGCGCCATTG |  |  |
| d_mas_F2_Fw | | GCATTGCTGGCCTGACCCACAACGAC | MSMEG_4727 fragment (flank_2) amplification to SLIC reaction (Δ*mas* strain) | |
| d_mas_F1_BamH_Rv | | TGACACTATAGAATACATAGGATCCCCCGCTTCGCAGC |  |  |
| 1061-60_**Hind**_SLIC_Fw | | GTGATAAACTACCGCATTA**AAGCTT**ATGCGCCAAGAGGTCACC | MSMEG_1061-1060 (flank_1) amplification to SLIC reaction (*MSMES_1060-mTurquoise* strain) | |
| 1061-60_**Kpn**_add_SLIC_Rv | | CCGACGACCT**GGTACC**CACCCTCAACTGCGA |  |  |
| 1059_Kpn_add_SLIC_Fw | | GGTG**GGTACC**AGGTCGTCGGCGCG | MSMEG_1059 (flank_2) amplification to SLIC reaction (*MSMES_1060-mTurquoise* strain) | |
| 1059_Eco52I-out_SLIC_Rv | | AGGGGAATTCTTAATTAAGCTCACCGCCGAGGTTGACG |  |  |
| L2-mTurquoise2-Fw | | GGCTCGGCGGGCTCGGCGGCGGGCTCGGGCGAGTTCATGGTCTCCAAGGGCGAGGAGC | Linker addition to *mTurquoise2* | |
| mTurquoise2-Stop-Kpn-Rv | | ATGGGTACCTCACTTGTACAGCTCG |  |  |
| mT2_1060_SLIC_Fw | | TCGCAGTTGAGGGTGGGTACCGGCTCGGCGGGCT | *mTurquoise2* with linker amplification to SLIC reaction  (*MSMES_1060-mTurquoise* strain) | |
| mT2_1060_Kpn_SLIC_Rv | | ACGCGCCGACGACCT**GGTACC**ATG**GGTACC**TCACTTGTACAGCT |  |  |
| Flag_oligo_Fw_**PmlI**/BamH | | **GTG**GACTACAAGGACGATGACGACAAGGACTACAAGGACGATGACGACAAGGACTACAAGGACGATGACGACAAG*TGA*G | FLAG sequence with *START codon*, oligo pair to hybridization | |
| Flag_oligo_Rv_**PmlI**/BamH | | GATCC*TCA*CTTGTCGTCATCGTCCTTGTAGTCCTTGTCGTCATCGTCCTTGTAGTCCTTGTCGTCATCGTCCTTGTAGTC**CAC** |  |  |
| RT-mas2_Fw | | CCAAGGTCTACCGCATGGC | analysis of *MSMEG_4727* gene expression by RT-qPCR | |
| RT-mas2_Rv | | CCAGGCTGGAGAACTCGAG |  |  |
| RT-lsr2_Fw | | CGATGATTTCGACGGTGAG | analysis of *lsr2* gene expression by RT-qPCR | |
| RT-lsr2_Rv | | ACCCACTGCTTCAGATCGTT |  |  |
| RT-1060_Fw | | GCTACATCAAGGCCGCAAAG | analysis of *MSMEG_1060* gene expression by RT-qPCR | |
| RT-1060_Rv | | GTTCGAGGTCCCGTTCGATT |  |  |
| GC_msmeg_FW | | GCAGCGCCCCGCCGACAT | amplification of *M. smegatis* chromosomal DNA fragment containing 73% GC pairs | |
| GC_msmeg_RV | | CTGCTGTGCTGTTCGGCTGCACGTA |  |  |
| AT_msmeg_FW | | ACTGGCGTGATCTGAGCGTT | amplification of *M. smegatis* chromosomal DNA fragment containing 57% GC pairs | |
| AT_msmeg_RV | | TCCCGGGCACCGACCCTT |  |  |
| NIR700_pTZ_FW | | TCGGTACCTCGCGAATGCATC | 5’-near-infrared-labeled primers for amplification of DNA fragments cloned into pTZ57R/T vector | |
| NIR700_pTZ_RV | | ATGCAGGCCTCTGCAGTCGAC |  |  |

References

1. Kołodziej M, Trojanowski D, Bury K, Hołówka J, Matysik W, Kąkolewska H, Feddersen H, Giacomelli G, Konieczny I, Bramkamp M, Zakrzewska-Czerwińska J. 2021. Lsr2, a nucleoid-associated protein influencing mycobacterial cell cycle. Sci Rep 11:2910.

2. Parish T, Stoker NG. 2000. Use of flexible cassette method to generate a double unmarked Mycobacterium tuberculosis tlyA plcABC mutant by gene replacement. Microbiology 146:1969–1975.
