# Supplementary material for: Lsr2 and Its Novel Paralogue Mediate the Adjustment of Mycobacterium smegmatis to Unfavorable Environmental Conditions
Source: mSphere. 2021 May 12;6(3):e00290-21. doi: 10.1128/mSphere.00290-21 (PMC8125055; doi:10.1128/mSphere.00290-21)
Supplement: TEXT S1 [file mSphere.00290-21-sd001.docx]

**Supplemental Experimental Procedures**

***M. smegmatis* mc^2^ 155 mutant strains construction**

The allelic replacement of gene encoding Lsr2 (MSMEG_6092) protein with *lsr2-flag3*, and MSMEG_1060 to *MSMEG_1060-mTurquoise2* was performed accordingly to Parish and Stoker (1). In the case of MSMEG_1060 fluorescence fusion, flanking sequences containing the *MSMEG_1059* and *MSMEG_1060-61* genes were amplified using as a template *M. smegmatis* mc^2^ 155 (WT) chromosomal DNA and primer pairs: 1059_Kpn_add_SLIC_Fw/1059_Eco52I-out_SLIC_Rv and 1061-60_Hind_SLIC_Fw/1061-60_Kpn_add_SLIC_Rv (Table S6). PCR products were cloned to p2NIL vector (kan*^R^*) using SLIC (Sequence and Ligation Independent Cloning) (2). In the case of Lsr2-Flag_3_ fusion, three times repeated FLAG sequence with BamHI and PmlI overhangs was prepared by hybridization of oligo pair Flag_oligo_Fw_PmlI/ Flag_oligo_Rv_PmlI/BamH and then cloned to p2NIL derivative containing upstream and downstream region of *lsr2* gene (3).

For construction of *M. smegmatis* mc^2^ 155 strain with deletion of *MSMEG_1060* gene cloning strategy was similar to creating MSMEG_1060-mTurquoise2 (see above) but flanking sequences containing the *MSMEG_1060* upstream and downstream region were amplified using primer pairs Ms_1061_Slic_Fw/Rv and Ms_1059_Slic_Fw/Rv (Table. S6).

For construction of Δ*mas* *M. smegmatis* mc^2^ 155 strain, only the promotor region (122 bp downstream) and first 378 bp of *MSMEG_4727* gene was deleted from *M. smegmatis* chromosome. Flanking sequences containing upstream and downstream region of deleted fragment were amplified using primer pairs d_mas_F1_Hind_Fw/d_mas_F1_Rv and d_mas_F2_Fw/d_mas_F1_BamH_Rv (Table S6).

All p2NIL derivatives were verified by sequencing (Microsynth). At the end, the pGoal17 cassette was cloned into the PacI site of each p2NIL derivative. *M. smegmatis* cells were then transformed with 200-1000 ng of NaOH/EDTA-treated plasmid DNA and unmarked mutants were selected accordingly to the procedure described previously (1). Mutants were analyzed by PCR, DNA sequencing and/or Western blotting. RT-qPCR was performed to confirm disruption of MSMEG_4727 gene in Δ*mas* (Fig. 2B main text)*.*

**DNA manipulations, bacterial strains, and culture conditions**

Bacterial strains construction and cultures was performed as described previously (3). Briefly, plasmids used for *M. smegmatis* mc^2^ 155 transformation were reproduced in the *E. coli* DH5α strain. *E. coli* was grown in LB broth or agar plates (Difco) supplemented by ampicillin (100 µg/ml) or kanamycin (50 µg/ml), accordingly to standard procedures (4). *M. smegmatis* strains were grown in 7H9 broth supplemented with 10% OADC (oleic acid-albumin-dextrose-catalase; BD) and 0.05% Tween 80, or on NB agar plates (Difco) supplemented with X-Gal (5-bromo-4-chloro-3-indolyl-α-D-galactopyranoside), 2% sucrose and/or kanamycin (50 µg/ml). Enzymes and reagents were provided by Thermo Fisher, Roth, and Merck (Sigma-Aldrich). Oligonucleotides were synthesized by Merck (Sigma-Aldrich), and sequencing was performed by Microsynth. To determine the growth curve for *M. smegmatis* strains in optimal conditions and during exposure to antibiotics, cells were grown at 37˚C in a final volume of 300 μl 7H9 (supplemented with OADC and Tween80), and optical density measurements were taken at 20-min intervals for 1-3 days using a Bioscreen C instrument (Growth Curves). Rapid anaerobic dormancy (RAD) model was used to shift *M. smegmatis* cultures from aerobic growth to hypoxia (5–7). 40 ml of liquid cultures (OD_600_ ~ 0.3) in tightly closed plastic tubes (maximum volume 50 ml) was incubated in 37˚C, with 180 rpm shaking for 6 hours. To optimize RAD model, oxygen consumption in cultures was monitored by methylene blue decolorization (5). Methylene blue (6 µg/ml) was added to cultures before they were sealed. The optical density at 600 nm was measured every hour for 10 hours but after 6 hours cultures were already decolorized.

**Protein purification**

The Lsr2_His6_ and MS1060_His6_ proteins were purified using nickel-affinity chromatography (personal communication with dr Trojanowski, unpublished data). Briefly, cells from 1.6 liter culture were lysed by sonication in 50 ml of LBA buffer (50 mM Tris-HCl [pH 7.5], 150 mM NaCl, 10 mM imidazole) and cellular extract was clarified by centrifugation (25000 g, 45 min, 4 °C). Clarified lysate was incubated

(4 °C, 16 h) with 1 ml bed volume of Ni-NTA agarose resin (Qiagen). The resin was washed tree times with LBA buffer and bound proteins were eluted with LBB and LBC buffers (buffer LBA supplemented with 125 mM imidazole and 250 mM imidazole, respectively).

**DNA binding assay**

EMSA (electrophoretic mobility shift assay) experiments were carried out as described previously (8), with some modifications. The 167-bp (73% GC pairs) and 116-bp (57% GC pairs) DNA fragments were amplified with appropriate primers (Table S6) and cloned into pTZ57R/T vector (Thermo Scientific). The 230-bp (70% GC pairs) and 179-bp (50% GC pairs) DNA fragments were amplified using a pair of near-infrared-labeled primers (Table S6). The increasing amounts of Lsr2_His6_ and MSMEG_1060_His6_ proteins were incubated with 50 fmoles of both DNA fragments for 15 min at 20°C in DNA binding buffer (50 mM Tris-HCl [pH 7.5], 150 mM NaCl, 10 mM magnesium acetate, 0.02% Tween-20, 5% [vol/vol] glycerol, 1.0 mg/ml bovine serum albumin) in a final volume of 20 μl. The reaction products were subjected to 5% native PAGE in 1x TBE buffer at 4°C for 6 h. Near-infrared signals were detected using an Azure c600 imaging system (Azure Biosystems).

**Atomic force microscopy (AFM)**

To estimate the roughness profile, *M. smegmatis* cultures were grown in 7H9-OADC-Tween 80 medium, centrifuged (6000 rpm for 5 minutes), washed with PBS and water, smeared onto microscopic slides, and dried. AFM measurements were conducted using the PeakForce Tapping mode (scan rate, 1.5 Hz; amplitude, 100 nm) on a BioScope Resolve AFM microscope (Bruker). ScanAsyst-Fluid+ cantilevers (Bruker) were used for AFM scanning (resonant frequency, 150 kHz; spring constant, 0.7 N/m). The Ra parameter (arithmetic mean deviation of the roughness profile) was calculated from the cell surface area using the NanoScope Analysis 1.9 software (Bruker).

**RNA isolation**

For RT-qPCR reaction and total RNA sequencing (RNA-seq), RNA was isolated with Trizol LS reagent (Invitrogen) as described previously (9, 10). Briefly, 20-40 ml of *M. smegmatis* culture was centrifuged at 5,000×g for 5 min at 4°C and next the cells were resuspended in 300 µl of water and lysed by bead-beating with the MP FastPrep system (MP Biomedicals) using the program: 2 × 45 s, 6.0 m/s with 5 min intervals on ice. RNA was purified and treated with DNase I (RapidOut DNA Removal Kit, Invitrogen) according to the manufacturer’s protocol. RNA quantity and integrity were checked by electrophoresis using an agarose gel, or/and by running the sample on an Agilent 2100 BioAnalyzer (Agilent RNA 6000 Nano Kit).

**Reverse-Transcription and Quantitative PCR (RT-qPCR)**

A total of 500 ng of RNA was used for cDNA synthesis using the Maxima First Strand cDNA synthesis kit (Thermo Fisher Scientific) in a final volume of 20 μl. The original manufacturer protocol was modified for GC-rich *M. smegmatis* transcripts by extending the first-strand synthesis time up to 30 min and increasing the temperature for synthesis to 65°C. Subsequently, the resulting cDNA was diluted (1:5) and used for quantitative PCRs performed with PowerUp SYBR Green Master Mix (Applied Biosystems). The relative level of a particular transcript was quantified using the comparative ΔΔCt method and the *sig*A gene as the endogenous control (StepOne Plus real-time PCR system, Applied Biosystems). The optimized oligonucleotides used in this study were synthetized by Sigma-Aldrich (Table S6).

**Extraction of lipids and TLC analysis**

Total lipids were extracted from dry cell mass of the wild type, Δ*lsr2*, *Δmas*, *Δlsr2Δmas,* and Δ*lsr2* complemented *M. smegmatis* strains according to the standard protocols (10). Briefly, the cells were dissolved in water and extracted twice with chloroform-methanol (1:2, v/v). Combined extracts were partitioned between chloroform/methanol and water, and then the organic phase was dried. Lipids were dissolved in small volume of chloroform and next precipitated in cold methanol (12).

Mycolic acids were obtained from delipidated cells (cells after chloroform-methanol extraction) by alkaline method using 15% tetrabutylamonium hydroxide, and then analyzed by TLC (13).

The lipid extracts were dissolved in chloroform-methanol (1:1, v/v) or chloroform in concentration of 50 mg/ml and applied on silica gel 60-precoated HPTLC or TLC plates (Merck, layer thickness 0.2 mm) with the same amount. Lipids were analyzed by TLC in different solvent systems: chloroform-methanol (9:1, v/v) for GPLs and LOSs; chloroform-methanol-water (60:30:6, v/v/v) for trehalose mycolates and phospholipids, hexane-diethyl ether (85:15, v/v) for mycolic acid methyl esters.

Sugar-containing compounds (GPLs, LOSs, trehalose dimycolates, trehalose monomycolates and phosphatidylinositol mannosides) were visualized by spraying the plates with 0.2% anthrone in concentrated sulfuric acid, or 0.5 % orcinol (13) followed by heating at 120°C. Mycolic acid methyl esters were visualized by 10% molybdophosphoric acid in ethanol solution, followed by heating at 120°C.

1. Parish T, Stoker NG. 2000. Use of flexible cassette method to generate a double unmarked Mycobacterium tuberculosis tlyA plcABC mutant by gene replacement. Microbiology 146:1969–1975.

2. Jeong JY, Yim HS, Ryu JY, Lee HS, Lee JH, Seen DS, Kang SG. 2012. One-step sequence-and ligation-independent cloning as a rapid and versatile cloning method for functional genomics Studies. Appl Environ Microbiol 78:5440–5443.

3. Kołodziej M, Trojanowski D, Bury K, Hołówka J, Matysik W, Kąkolewska H, Feddersen H, Giacomelli G, Konieczny I, Bramkamp M, Zakrzewska-Czerwińska J. 2021. Lsr2, a nucleoid-associated protein influencing mycobacterial cell cycle. Sci Rep 11:2910.

4. Sambrook J, Fritsch EF, Maniatis T. 1989. Molecular Cloning: A Labortaroy ManualSociety.

5. Leistikow RL, Morton RA, Bartek IL, Frimpong I, Wagner K, Voskuil MI. 2010. The Mycobacterium tuberculosis DosR regulon assists in metabolic homeostasis and enables rapid recovery from nonrespiring dormancy. J Bacteriol 192:1662–1670.

6. Reichlen MJ, Leistikow RL, Scobey MS, Born SEM, Voskuil MI. 2017. Anaerobic Mycobacterium tuberculosis cell death stems from intracellular acidification mitigated by the DosR regulon. J Bacteriol 199:320–337.

7. Bartek IL, Woolhiser LK, Baughn AD, Basaraba RJ, Jacobs WR, Lenaerts AJ, Voskuil MI, Cumming BM, Lamprecht D a, Wells RM, Saini V, Mazorodze JH, Steyn AJC, Voskuil MI, Schnappinger D, Visconti KC, Harrell MI, Dolganov GM, Sherman DR, Schoolnik GK, Macmicking J, Xie Q, Nathan CF, Darwin KH, Ehrt S, Gutierrez-Ramos J-C, Weich N, Nathan CF, Schnappinger D, Ehrt S, Voskuil MI, Liu Y, Mangan JA, Monahan IM, Dolganov GM, Efron B, Butcher PD, Nathan CF, Schoolnik GK, Bartek IL, Visconti KC, Schoolnik GK, Kumar A, Farhana A, Guidry L, Saini V, Hondalus M, Steyn AJC, Lamichhane G, Cunningham-bussel A, Zhang T, Nathan CF, Shell SS, Prestwich EG, Baek S-H, Shah RR, Sassetti CM, Dedon PC, Fortune SM, Fisher M a., Plikaytis BB, Shinnick TM. 2011. Mycobacterium tuberculosis Lsr2 Is a Global Transcriptional Regulator. Front Microbiol 2:e01106–e01114.

8. Wolański M, Donczew R, Kois-Ostrowska A, Masiewicz P, Jakimowicz D, Zakrzewska-Czerwińska J. 2011. The level of AdpA directly affects expression of developmental genes in Streptomyces coelicolor. J Bacteriol 193:6358–6365.

9. Pawelczyk J, Brzostek A, Kremer L, Dziadek B, Rumijowska-Galewicz A, Fiolka M, Dziadek J. 2011. AccD6, a Key Carboxyltransferase Essential for Mycolic Acid Synthesis in Mycobacterium tuberculosis, Is Dispensable in a Nonpathogenic Strain †. J Bacteriol 193:6960–6972.

10. Płociński P, Macios M, Houghton J, Niemiec E, Płocińska R, Brzostek A, Słomka M, Dziadek J, Young D, Dziembowski A. 2019. Proteomic and transcriptomic experiments reveal an essential role of RNA degradosome complexes in shaping the transcriptome of Mycobacterium tuberculosis. Nucleic Acids Res 47:5892–5905.

11. Burdon RH. Laboratory Techniques in Biochemistry and Molecular Biology.

12. Villeneuve C, Etienne G, Abadie V, Montrozier H, Bordier C, Laval F, Daffe M, Maridonneau-Parini I, Astarie-Dequeker C. 2003. Surface-exposed glycopeptidolipids of Mycobacterium smegmatis specifically inhibit the phagocytosis of mycobacteria by human macrophages: Identification of a novel family of glycopeptidolipids. J Biol Chem 278:51291–51300.

13. Schumann P. 1995. Chemical Methods in Prokaryotic Systematics. J Basic Microbiol 35:122–122.

14. Mordarska H, Pa~ciak M. 1994. A simple method for differentiation of Propionibacterium acnes and Propionibacterium propionicumFEMS Microbiology Letters.
