# Supplementary material for: Lsr2 and Its Novel Paralogue Mediate the Adjustment of Mycobacterium smegmatis to Unfavorable Environmental Conditions
Source: mSphere. 2021 May 12;6(3):e00290-21. doi: 10.1128/mSphere.00290-21 (PMC8125055; doi:10.1128/mSphere.00290-21)
Supplement: TABLE S2 [file mSphere.00290-21-st002.docx]

| **Table S2.** **Gene expression during hypoxia** | | | | |
| --- | --- | --- | --- | --- |
| **The altered expression of selected genes during hypoxia in WT M. smegmatis strain.** | | | | |
| gene | **Annotation** | | **FC** | |
| Transcriptional Regulator | | | | |
| **MSMEG_1178** | transcriptional regulator | | 5.4 | |
| **MSMEG_4768** | transcriptional regulator | | 2.9 | |
| **MSMEG_6605** | transcriptional regulatory protein | | 2.8 | |
| **MSMEG_6292** | transcription elongation factor GreA | | 2.6 | |
| **MSMEG_3264** | transcriptional regulator | | 2.4 | |
| **MSMEG_4300** | transcription regulator AmtR | | 2.3 | |
| **MSMEG_0742** | transcriptional regulatory protein | | 2.3 | |
| **MSMEG_0691** | transcriptional regulatory protein | | 2.0 | |
| **MSMEG_5025** | transcriptional regulator | | 1.9 | |
| **MSMEG_0213** | transcriptional regulator | | 1.8 | |
| **MSMEG_1919** | transcription factor WhiB | | 1.8 | |
| **MSMEG_3360** | transcriptional regulator | | 1.7 | |
| **MSMEG_1168** | transcriptional regulator | | 1.6 | |
| **MSMEG_6556** | transcriptional regulator | | 1.6 | |
| **MSMEG_5301** | transcriptional regulator | | -1.9 | |
| **MSMEG_0092** | transcriptional regulatory protein | | -2.3 | |
| **MSMEG_0778** | GntR family transcriptional regulator | | -2.3 | |
| **MSMEG_3092** | transcriptional regulator, sugar-binding family protein | | -3.3 | |
| **MSMEG_0155** | transcriptional regulator | | -5.3 | |
| RNA polymerase sigma factors | | | | |
| **MSMEG_1418** | RNA polymerase sigma-70 factor, ECF subfamily | | 2.2 | |
| **MSMEG_1690** | RNA polymerase sigma-70 factor, ECF subfamily | | 2.0 | |
| **MSMEG_1666** | RNA polymerase sigma factor SigJ, ECF subfamily | | 1.9 | |
| **MSMEG_0573** | RNA polymerase sigma-70 factor, RpoE1, ECF subfamily | | -1.5 | |
| **MSMEG_1804** | RNA polymerase sigma factor SigF | | -1.9 | |
| **MSMEG_1747** | RNA polymerase sigma-70 factor, ECF subfamily | | -2.2 | |
| Two-components system proteins | | | | |
| **MSMEG_3944** | two-component protein, DosR homolog | | 3.7 | |
| **MSMEG_5244** | DevR family transcriptional regulator | | 1.1 | |
| **MSMEG_0786** | serine/threonine-protein kinase PknG | | 3.3 | |
| **MSMEG_0529** | serine/threonine-protein kinase PknK | | 2.8 | |
| **MSMEG_0981** | two-component system regulator | | 2.1 | |
| **MSMEG_2807** | two-component system response regulator | | 1.9 | |
| **MSMEG_4968** | two-component system sensor kinase | | 1.6 | |
| **MSMEG_0244** | two-component response protein PrrA | | -1.6 | |
| **MSMEG_1200** | serine/threonine protein kinase | | -1.9 | |
| **MSMEG_6236** | two-component system, regulatory protein | | -3.7 | |
| **MSMEG_6238** | two-component system sensor kinase | | -3.9 | |
|  | | | | |
| **The altered genes expression in Δ*lsr2* strain compared to WT during hypoxia.** | | | | |
| gene | | **Annotation** | | **FC** |
| **MSMEG_1128** | | hypothetical protein | | 5.9 |
| **MSMEG_1129** | | D-amino-acid dehydrogenase | | 4.8 |
| **MSMEG_3374** | | hypothetical protein | | 4.7 |
| **MSMEG_1240** | | hypothetical protein | | 4.6 |
| **MSMEG_6147** | | hypothetical protein | | 4.5 |
| **MSMEG_1130** | | hypothetical protein | | 4.5 |
| **MSMEG_5710** | | hypothetical protein | | 4.4 |
| **MSMEG_1241** | | hypothetical protein | | 4.0 |
| **MSMEG_5957** | | algD; GDP-mannose 6-dehydrogenase AlgD | | 3.5 |
| **MSMEG_5501** | | hypothetical protein | | 3.3 |
| **MSMEG_3579** | | transmembrane protein | | 3.3 |
| **MSMEG_0200** | | hypothetical protein | | 3.1 |
| **MSMEG_0842** | | hypothetical protein | | 3.1 |
| **MSMEG_5558** | | hypothetical protein | | 3.1 |
| **MSMEG_5461** | | hypothetical protein | | 3.1 |
| **MSMEG_5972** | | hypothetical protein | | 3.1 |
| **MSMEG_5460** | | hypothetical protein | | 2.8 |
| **MSMEG_4730** | | hypothetical protein | | 2.8 |
| **MSMEG_5958** | | hypothetical protein | | 2.8 |
| **MSMEG_1239** | | ISMsm4, transposase | | 2.8 |
| **MSMEG_1247** | | hypothetical protein | | 2.6 |
| **MSMEG_3565** | | hypothetical protein | | 2.6 |
| **MSMEG_5438** | | ksgA; dimethyladenosine transferase | | 2.6 |
| **MSMEG_2199** | | hypothetical protein | | 2.6 |
| **MSMEG_1238** | | type III restriction enzyme, res subunit | | 2.6 |
| **MSMEG_1223** | | hypothetical protein | | 2.6 |
| **MSMEG_4728** | | condensation domain-containing protein | | 2.5 |
| **MSMEG_3352** | | hypothetical protein | | 2.5 |
| **MSMEG_2822** | | pseudogene | | 2.5 |
| **MSMEG_3578** | | cyclase | | 2.5 |
| **MSMEG_4727** | | mycocerosic acid synthase | | 2.4 |
| **MSMEG_2828** | | IS1096, tnpA protein | | 2.4 |
| **MSMEG_0841** | | pseudogene | | 2.3 |
| **MSMEG_3754** | | hypothetical protein | | 2.2 |
| **MSMEG_4729** | | hypothetical protein | | 2.2 |
| **MSMEG_0611** | | para-nitrobenzyl esterase | | 2.2 |
| **MSMEG_1135** | | hypothetical protein | | 2.2 |
| **MSMEG_4032** | | zinc-binding alcohol dehydrogenase | | 2.2 |
| **MSMEG_5459** | | hypothetical protein | | 2.2 |
| **MSMEG_5709** | | hypothetical protein | | 2.2 |
| **MSMEG_4046** | | L-carnitine dehydratase acid-inducible protein F | | 2.2 |
| **MSMEG_3719** | | sodium/calcium exchanger protein | | 2.2 |
| **MSMEG_3566** | | FadD16 protein | | 2.1 |
| **MSMEG_1248** | | hypothetical protein | | 2.1 |
| **MSMEG_1253** | | hypothetical protein | | 2.0 |
| **MSMEG_4814** | | hypothetical protein | | 1.9 |
| **MSMEG_1868** | | hypothetical protein | | 1.9 |
| **MSMEG_4844** | | acyl-CoA dehydrogenase | | 1.9 |
| **MSMEG_0840** | | hypothetical protein | | 1.8 |
| **MSMEG_3729** | | katG; catalase/peroxidase | | 1.8 |
| **MSMEG_0384** | | rfbA; glucose-1-phosphate thymidylyltransferase | | 1.8 |
| **MSMEG_6717** | | oxidoreductase, Gfo/Idh/MocA family protein | | 1.8 |
| **MSMEG_5433** | | 4Fe-4S ferredoxin | | 1.8 |
| **MSMEG_2717** | | hypothetical protein | | 1.7 |
| **MSMEG_3695** | | hypothetical protein | | 1.7 |
| **MSMEG_5231** | | hypothetical protein | | 1.7 |
| **MSMEG_4785** | | mce-family protein mce1f | | 1.7 |
| **MSMEG_3577** | | calpastatin | | 1.7 |
| **MSMEG_4731** | | acyl-CoA synthetase | | 1.6 |
| **MSMEG_5093** | | IS1096, tnpR protein | | 1.6 |
| **MSMEG_4547** | | acyl-CoA dehydrogenase | | 1.6 |
| **MSMEG_4816** | | 6-phosphogluconate dehydrogenase | | 1.6 |
| **MSMEG_4363** | | hypothetical protein | | 1.6 |
| **MSMEG_0377** | | nitrile hydratase | | 1.6 |
| **MSMEG_0198** | | acyl-CoA dehydrogenase | | 1.6 |
| **MSMEG_2826** | | hypothetical protein | | 1.6 |
| **MSMEG_2149** | | hypothetical protein | | 1.6 |
| **MSMEG_1470** | | rplF; 50S ribosomal protein L6 | | -1.6 |
| **MSMEG_1474** | | rplO; 50S ribosomal protein L15 | | -1.6 |
| **MSMEG_4637** | | hypothetical protein | | -1.6 |
| **MSMEG_4840** | | Rieske (2Fe-2S) domain-containing protein | | -1.6 |
| **MSMEG_0065** | | hypothetical protein | | -1.6 |
| **MSMEG_6894** | | rplI; 50S ribosomal protein L9 | | -1.6 |
| **MSMEG_1525** | | 50S ribosomal protein L17 | | -1.6 |
| **MSMEG_1448** | | integral membrane transporter; SulP family | | -1.6 |
| **MSMEG_1467** | | rplE; 50S ribosomal protein L5 | | -1.6 |
| **MSMEG_0408** | | type I modular polyketide synthase | | -1.6 |
| **MSMEG_6880** | | hydrophobic amino acid ABC transporter | | -1.7 |
| **MSMEG_6691** | | glutamine amidotransferase; | | -1.7 |
| **MSMEG_1472** | | rpsE; 30S ribosomal protein S5 | | -1.7 |
| **MSMEG_6638** | | metE | | -1.7 |
| **MSMEG_0340** | | enoyl-CoA hydratase/isomerase | | -1.7 |
| **MSMEG_0781** | | amino acid permease | | -1.7 |
| **MSMEG_3970** | | glutamyl-tRNA(Gln) amidotransferase subunit A | | -1.7 |
| **MSMEG_6115** | | phosphoglycerate dehydrogenase | | -1.7 |
| **MSMEG_1524** | | rpoA; DNA-directed RNA polymerase subunit alpha | | -1.7 |
| **MSMEG_1439** | | rplB; 50S ribosomal protein L2 | | -1.8 |
| **MSMEG_4815** | | hypothetical protein | | -1.8 |
| **MSMEG_1437** | | rplD; 50S ribosomal protein L4 | | -1.8 |
| **MSMEG_1468** | | rpsN; 30S ribosomal protein S14 | | -1.8 |
| **MSMEG_1459** | | Rieske (2Fe-2S) domain-containing protein | | -1.8 |
| **MSMEG_1143** | | mce related protein | | -1.8 |
| **MSMEG_1444** | | rpmC; 50S ribosomal protein L29 | | -1.8 |
| **MSMEG_3499** | | hypothetical protein | | -1.9 |
| **MSMEG_0354** | | hypothetical protein | | -1.9 |
| **MSMEG_0911** | | aceA; isocitrate lyase | | -1.9 |
| **MSMEG_1443** | | rplP; 50S ribosomal protein L16 | | -1.9 |
| **MSMEG_2166** | | transketolase | | -1.9 |
| **MSMEG_4425** | | oxidoreductase | | -2.0 |
| **MSMEG_0150** | | NAD(P) transhydrogenase subunit beta | | -2.0 |
| **MSMEG_1690** | | ECF sigma factor RpoE1 | | -2.0 |
| **MSMEG_1365** | | rplL; 50S ribosomal protein L7/L12 | | -2.0 |
| **MSMEG_1469** | | rpsH; 30S ribosomal protein S8 | | -2.0 |
| **MSMEG_6261** | | glutamine amidotransferase | | -2.0 |
| **MSMEG_1438** | | rplW; 50S ribosomal protein L23 | | -2.0 |
| **MSMEG_5419** | | pseudogene | | -2.1 |
| **MSMEG_5334** | | hypothetical protein | | -2.1 |
| **MSMEG_1401** | | tuf; elongation factor Tu | | -2.1 |
| **MSMEG_4174** | | IclR family transcriptional regulator | | -2.1 |
| **MSMEG_6297** | | aldehyde dehydrogenase | | -2.2 |
| **MSMEG_0286** | | GntR family transcriptional regulator | | -2.2 |
| **MSMEG_1441** | | rplV; 50S ribosomal protein L22 | | -2.2 |
| **MSMEG_2165** | | transketolase | | -2.2 |
| **MSMEG_1442** | | rpsC; 30S ribosomal protein S3 | | -2.3 |
| **MSMEG_6458** | | gltD; glutamate synthase subunit beta | | -2.3 |
| **MSMEG_2157** | | hypothetical protein | | -2.3 |
| **MSMEG_0143** | | mce associated membrane protein | | -2.3 |
| **MSMEG_2155** | | trans-2-enoyl-CoA reductase | | -2.3 |
| **MSMEG_1440** | | rpsS; 30S ribosomal protein S19 | | -2.6 |
| **MSMEG_3701** | | hypothetical protein | | -2.7 |
| **MSMEG_4835** | | 3-oxoacyl-ACP reductase | | -2.7 |
| **MSMEG_6878** | | inner-membrane translocator | | -3.2 |
| **MSMEG_6092** | | Lsr2 protein | | -3.9 |
| **MSMEG_6093** | | hypothetical protein | | -5.1 |
